# Tunneling field emission from nano-optics under electron irradiation

**DOI:** 10.1126/sciadv.ady5421

**Published:** 2026-01-02

**Authors:** Kenan Elibol, Vesna Srot, Chao Yang, Sayooj Satheesh, Serkan Arslan, Marko Burghard, Harald Giessen, Peter A. van Aken

**Affiliations:** ^1^ Max Planck Institute for Solid State Research, Heisenbergstr. 1, 70569 Stuttgart, Germany.; ^2^ 4th Physics Institute and Research Center SCoPE, University of Stuttgart, 70569 Stuttgart, Germany.

## Abstract

While field emission, which is crucial for sub–angstrom resolution imaging and nanofabrication, has been extensively investigated in high-voltage and laser-driven systems, its realization through plasmonics under electron irradiation remains unexplored. Here, we demonstrate a proof of concept for electron beam–driven field emission from plasmonic emitter arrays with nanoscale hotspots acting as highly efficient emission sites, which also exhibit strong surface-enhanced Raman scattering activity. The electron beam–induced field distributions and electrical polarization switching are visualized by four-dimensional scanning transmission electron microscopy. Classical and quantum-corrected electromagnetic simulations combined with experimental field maps identify the highest field intensities at the apex of emitter tips. Optical excitation experiments confirm the giant field enhancement at these locations. In situ electrical measurements in a transmission electron microscope reveal a distinct tunneling field emission signature that surpasses secondary electron emission. The demonstrated field emission mechanism opens unexplored possibilities for realizing spatially confined electron sources with ultrafast temporal switching capabilities.

## Teaser

Electron irradiation of lithographically defined metal nanostructures on conductive substrates enables tunneling field emission.

## INTRODUCTION

The discovery of the photoelectric effect in 1887 and its theoretical explanation in 1905 laid the foundation for quantum mechanics, driving pioneering research on photoexcited electrons and their role in various physical and chemical processes (*1*–*3*). In 1928, Fowler-Nordheim (FN) tunneling, a quantum mechanical phenomenon, provided a fundamental understanding of field electron emission from metals (*4*). Since then, field emission has attracted notable interest because of its diverse applications in electron microscopy, nanolithography, microwave amplifiers, and x-ray generation (*5*–*8*). This process occurs when conduction electrons tunnel through a potential barrier that is reduced under a strong external electric field, enabling their emission into a vacuum. Unlike photoemission, which is driven by photon absorption, FN tunneling enables a field-controlled electron emission mechanism (*9*). In plasmonic nanostructures, the distinction between photoemission and field emission is characterized by the Keldysh parameter, defined as $\gamma =\omega \sqrt{2mW_{F}}/\mathrm{eE}$, where ω is the angular frequency, *m* is the electron mass, *W*_F_ is the work function of the surface, *e* is the elementary charge, and *E* is the amplitude of the enhanced electric field (*9*). In 1936, Malter (*10*) observed an additional electron emission mechanism, known as anomalous secondary electron (SE) field emission, by irradiating a thin insulating oxide-covered metal surface using an electron beam. While conventional field emission occurs via FN tunneling without prior energy transfer, SE field emission is initiated by energy transfer from incident electrons to bound electrons (*11*). Despite these historical achievements, experimental verification of FN field emission from conduction electrons in metals under electron irradiation remains elusive. This is a critical gap because understanding electron emission from electron-irradiated materials is essential for practical applications in electron microscopy and lithography. In addition, with recent advancements in attomicroscopy (*12*), electron beam–driven field emission could provide a powerful means to investigate time-resolved field emission dynamics in plasmonic systems excited by attosecond electron pulses (*13*, *14*).

Advancements in ultrafast laser technology have enabled the generation of intense electric fields capable of directly inducing field emission from surfaces (*7*, *15*–*17*). According to FN theory, the number of field-emitted conduction electrons increases exponentially with the applied field strength (*18*). This effect is further enhanced in plasmonic systems, where the strong localization of evanescent fields at nanoscale hotspots can substantially amplify electron emission (*9*, *19*–*23*). In coupled plasmonic nanostructures with subnanometer gaps, extreme field confinement at these hotspots enables FN tunneling at substantially lower external field strengths, reducing the need for high-intensity driving laser fields (*24*–*27*). While FN tunneling in plasmonic systems can be indirectly inferred by analyzing the gap volume where plasmon energy is concentrated in coupled metal nanoparticles (*28*), the direct observation of tunneling field emission from electron beam–irradiated plasmonic nanostructures remains unachieved. This limitation arises from experimental constraints and the absence of suitable emitter architectures.

Here, we address these challenges and present direct experimental evidence of electron beam–driven field emission from plasmonic nanostructures exposed to an intense electron beam. Our fabricated plasmonic emitter arrays, featuring strong plasmonic hotspots, serve as efficient electron emission sites under 200-kV parallel electron-beam irradiation and exhibit highly effective surface-enhanced Raman scattering (SERS) activity. To characterize the electron beam–induced electric field distributions, we use four-dimensional scanning transmission electron microscopy (4D-STEM) and validate the results through finite element method (FEM) simulations. 4D-STEM is also used to visualize electrical polarization switching in coupled nanostructures subjected to external electrical bias in the transmission electron microscope (TEM). The field enhancement at emitter hotspots is further confirmed via SERS measurements and electromagnetic simulations incorporating optical excitations. To establish a definitive FN tunneling signature from emitters under electron-beam irradiation, we conduct in situ TEM electrical measurements, revealing a field emission process that largely exceeds SE emission.

## RESULTS

### Electron-emitting plasmonic nanostructures

**Fig. 1.**
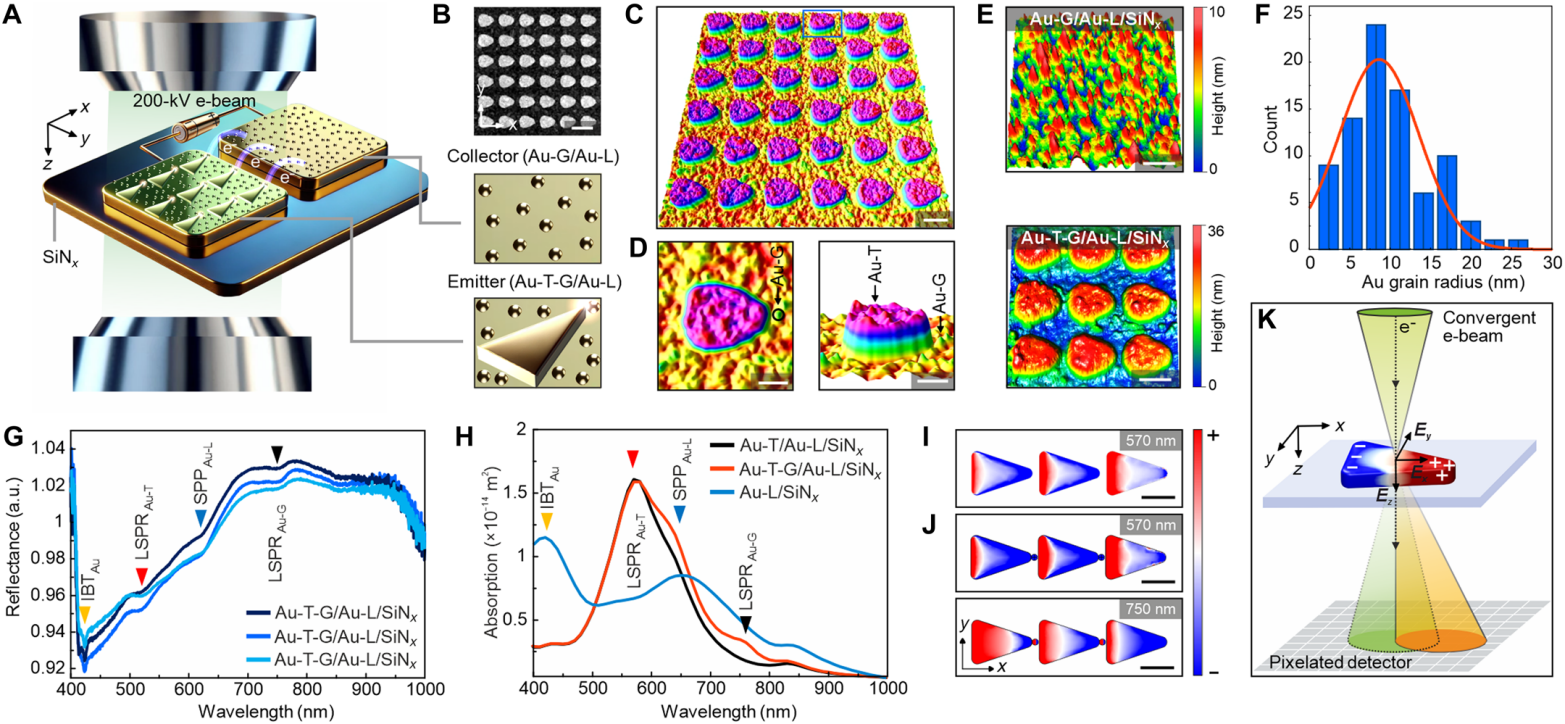
Overview of the experimental setup and emitter structures. (**A**) Schematic illustration of the experiment showing Au-T-G electron emitters under parallel electron-beam irradiation. Emitted electrons are collected by a positively biased electrode. (**B**) HAADF-STEM image of Au-Ts fabricated on a Au-L deposited onto a SiN*_x_* membrane. (**C**) Three-dimensional (3D) surface plot of the HAADF-STEM image in (B). (**D**) Close-up 3D surface plot showing the plane view and the cross section of the triangle marked in the blue frame in (C). (**E**) 3D AFM topography images of Au-G/Au-L/SiN*_x_* and Au-T-G/Au-L/SiN*_x_* structures. (**F**) Histogram showing the size distribution of hemispherical Au-Gs. (**G**) Reflectance spectra measured on Au-T-G/Au-L/SiN*_x_*. a.u., arbitrary units. (**H**) Computed absorption spectrum for Au-T/Au-L/SiN*_x_*, Au-T-G/Au-L/SiN*_x_*, and Au-L/SiN*_x_*. The red and black arrows at 623 nm (1.99 eV) and 424 nm (2.92 eV) mark the dipole modes of Au-T and Au-G, while the blue and orange arrows mark the SPP and the interband transition (IBT) of Au, respectively. (**I** and **J**) Simulated surface eigencharge distributions obtained at different LSPR energies, which are identified from the absorption spectra of Au-T/Au-L/SiN*_x_* and Au-T-G/Au-L/SiN*_x_* in (H), respectively. (**K**) Schematic representation of the 4D-STEM setup used for mapping the electron beam–induced electric field distribution in Au-T/SiN*_x_* structures. Scale bars: 200 nm (B), 100 nm [(C), (E), (I), and (J), and 50 nm (D)].

Electron beam–driven field emission from plasmonic nanostructures under 200-kV parallel electron-beam irradiation is measured in situ within a TEM (Fig. 1A). The emitter arrays consist of elongated gold (Au) triangles (Au-Ts) coupled with hemispherical Au grains (Au-Gs), all positioned on a prepatterned Au layer (Au-L) deposited onto a silicon nitride (SiN*_x_*) membrane (Fig. 1B and figs. S1 and S2). The surface plot of the high-angle annular dark-field (HAADF) image reveals that Au-Ts are coupled to Au-Gs, which are distributed across the Au layer (Fig. 1C). These Au-Gs, distributed around the Au-Ts—which are spaced 20 nm apart—are formed via thermal evaporation of Au onto the amorphous SiN*_x_* membranes at room temperature (Fig. 1, C to E, and fig. S3). A separate Au-L, free of emitters, serves as a collector, and it is positioned 3 μm away from the Au-T-G emitters. Emitted electrons are driven toward the collector under a static external electric field *E*^ext^ for current measurement. The Au-L contains intrinsic Au-Gs with a size distribution of 8.63 ± 0.8 nm and an areal density of 0.5/1000 nm^−2^ (Fig. 1F).

Under our parallel-beam TEM conditions (see Materials and Methods), the dose rate is ~2.46 e^−^ Å^−2^ s^−1^. Thus, a 60-s exposure yields a total dose of ~148 e^−^ Å^−2^, which is moderate for Au under parallel illumination. At 200 kV, knock-on displacement of Au is negligible (*29*), and the beam-assisted surface diffusion/heating is minimized by the large illuminated area. Consistent with this, we observe no measurable change in tip radius or gap width during acquisition (Fig. 1, C and D). We also limit the cumulative dose with short exposures and by avoiding repeated rescans. Thus, our illumination conditions are unlikely to increase Au surface mobility or alter the nanogap geometry in a way that affects our conclusions.

In Fig. 1G, the experimental reflectance spectra of Au-T-G/Au-L/SiN*_x_* exhibit localized surface plasmon resonance (LSPR) peaks at 520 nm (2.38 eV) and 746 nm (1.66 eV). These positions agree closely with the simulated absorption spectra (Fig. 1H), with minor shifts attributed to fabrication-induced variations in geometry and local dielectric environment. The surface eigencharge distributions (Fig. 1, I and J) reveal a bonding-type dipolar LSPR in the Au-T-G emitter structure, which comprises coupled Au-T and Au-G, whereas the isolated Au-T supports a conventional dipolar mode. To experimentally map the electron beam–induced electric field distribution, we perform 4D-STEM (Fig. 1K).

**Fig. 2.**
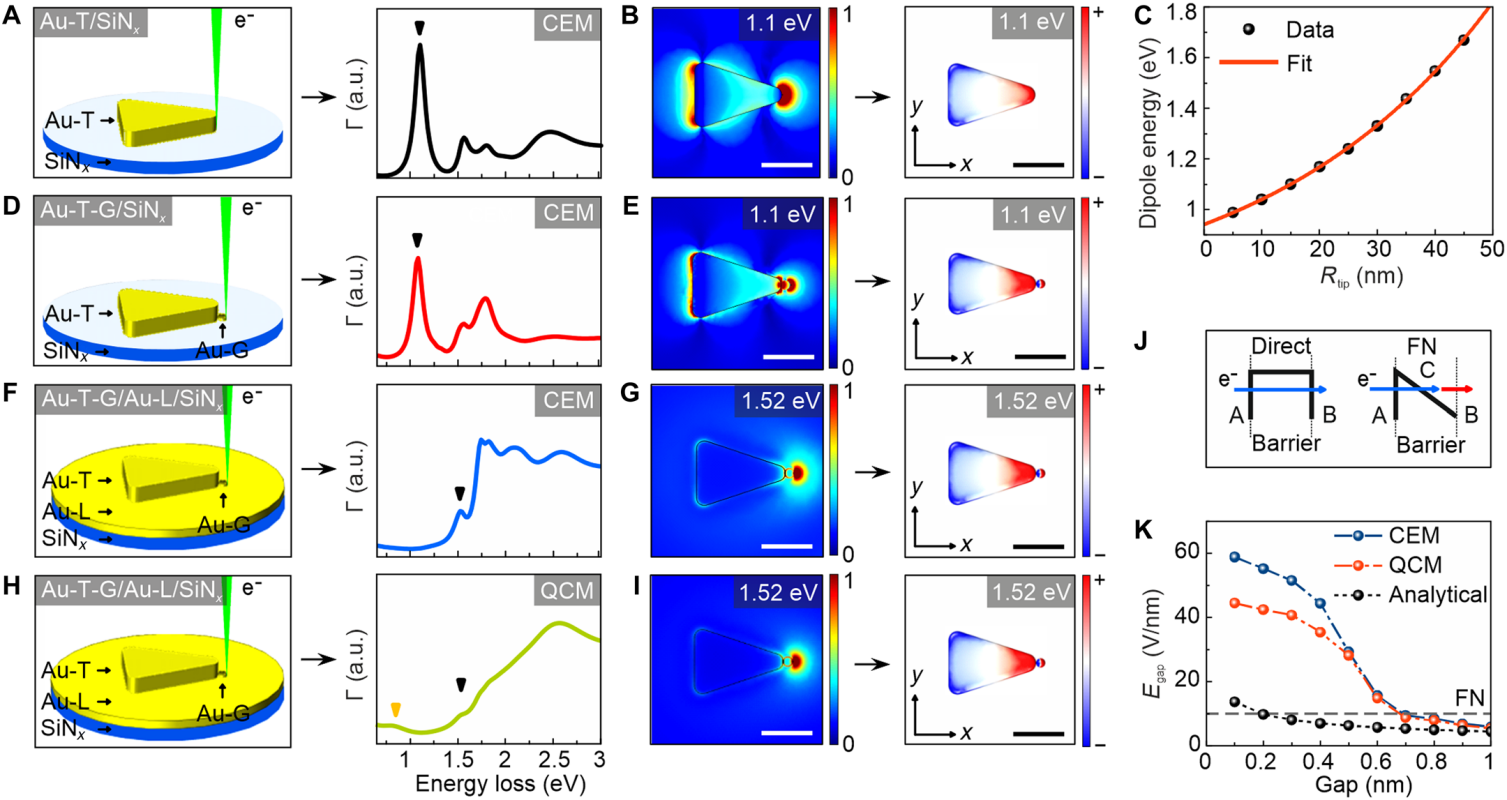
Classical and quantum-corrected electromagnetic simulations. (**A**) Schematic of the Au-T/SiN*_x_* model used in CEM simulations, along with the corresponding simulated EEL spectrum. (**B**) Simulated normalized electric field map and surface eigencharge distribution at the dipolar LSPR energy identified in the EEL spectrum in (A). (**C**) Dipolar LSPR energy of the Au-T/SiN*_x_* structure as a function of the tip radius. (**D**) Schematic of the Au-T-G/SiN*_x_* model used in CEM simulations, along with the corresponding simulated EEL spectrum. (**E**) Simulated normalized electric field map and surface eigencharge distribution at the dipolar LSPR energy identified in the EEL spectrum in (D). (**F**) Schematic of the Au-T-G/Au-L/SiN*_x_* model used in CEM simulations, along with the corresponding simulated EEL spectrum. (**G**) Simulated normalized electric field map and surface eigencharge distribution at the dipolar LSPR energy identified in the EEL spectrum in (F). (**H**) Schematic of the Au-T-G/Au-L/SiN*_x_* model used in QCM simulations, along with the corresponding simulated EEL spectrum. (**I**) Simulated normalized electric field map and surface eigencharge distribution at the dipolar LSPR energy identified in the EEL spectrum in (H). EEL spectra are derived from the positions where the electron beam is located in (A), (D), (F), and (H). The dipole modes are marked by black triangles in (A), (D), (F), and (H). (**J**) Schematics of the classical and FN tunneling processes. (**K**) Electric field strength calculated by the CEM, QCM, and analytical model. The gap between the Au-T tip and Au-G in the Au-T-G/Au-L/SiN*_x_* structure is varied from 0.1 to 1 nm. Scale bars: 100 nm [(B), (E), (G), and (I)].

The optical properties of individual emitter structures are revealed by electron energy loss (EEL) computed via FEM with the classical electromagnetic model (CEM). The tip radius (*R*_tip_) is 15 nm for the Au-T (fig. S4), while the radius of the hemispherical Au-G is 8.6 nm in the simulations. The Au-T structures support multiple LSPRs (Fig. 2A). The dominant dipolar LSPR is identified through simulated normalized electric field maps and surface eigencharge distributions at the corresponding plasmon energy (Fig. 2B). In addition to the primary dipolar LSPR at 1.1 eV, higher-order LSPRs with lower cross sections are also excited (fig. S5). The dipolar LSPR, exhibiting the highest cross section and strong localization at the Au-T tip, notably enhances electron emission from these regions. Notably, as the tip radius *R*_tip_ increases, the dipolar LSPR undergoes a blue shift (Fig. 2C).

When a Au-T couples with a neighboring Au-G, a bonding-type dipolar LSPR emerges (Fig. 2, D and E). Because of slight energy differences between the dipolar modes of Au-T and Au-G on SiN*_x_*, no large enhancement, spectral splitting, or energy shift is observed in the dipole mode of the Au-T (Fig. 2, A, B, D, and E, and fig. S6). However, when placed on a Au-L, the dipolar LSPR of Au-T blue-shifts to 1.52 eV, whereas that of Au-G red-shifts to 1.56 eV because of substrate-induced plasmonic interactions (Fig. 2, F and G, and fig. S6) (*30*). Given that the dipole energies of Au-T and Au-G become nearly identical on Au-L, stronger field enhancement can be achieved via dipole coupling.

In addition to simulations with the CEM, we also implement the quantum-corrected model (QCM) for electromagnetic simulations to reveal the field strength at coupled nanoparticles with subnanometer gaps (see Materials and Methods). Similar to the CEM, the QCM shows that the LSPR of the Au-T-G/Au-L/SiN*_x_* structure is at 1.52 eV (Fig. 2, H and I). However, we also observe an additional peak at 0.8 eV corresponding to a charge transfer plasmon, which can be formed as a result of direct electron transfer or tunneling (see surface eigencharge distribution in fig. S7). Given that EELS spectra and field profiles are essentially the same for ideal and nonideal models, we use ideal models in all simulations for computational simplicity (fig. S8).

The dipolar LSPRs measured by reflectance appear at higher energies compared to LSPRs obtained by EELS simulations from a single Au-T-G/Au-L/SiN*_x_* emitter. This is because of the following: (i) Reflectance probes far-field, radiative (bright) modes of an ensemble, whereas EELS excites near-field responses (including dark and out-of-plane components) of a single particle. (ii) For in-plane dipolar oscillations near a metal film, the image dipole opposes the particle dipole. This increases the restoring force and blue-shifts the bright dipolar resonance relative to the isolated particle. (iii) Reflectance is ensemble-averaged and weighted by the radiative cross section.

At subnanometer gaps, electron transport occurs either through direct tunneling, where electrons in region A tunnel directly through the potential barrier into region B, or via FN tunneling, where electrons first tunnel through a triangular barrier into region C (a vacuum gap) before reaching region B (Fig. 2J). Given that Au-Ts and hemispherical Au-Gs are electrically shorted by a continuous Au layer, their Fermi levels are equal. Although subnanometer tunneling can occur across the gap, any excess charge is screened over angstrom-scale lengths and drained through the film. As the metals do not sustain extended space-charge regions, only transient and localized dipoles can form unless the metallic elements are electrically isolated or biased. The SiN*_x_* membrane serves primarily as mechanical support and has a negligible impact on the charge distribution between triangles and grains.

The plasmon-enhanced electric field at the gap *E*_gap_ is estimated using the analytical model of $\omega_{D}=\left(\varepsilon_{0}/2\right){|E_{\text{gap}}|}^{2}V_{\text{gap}}$, where $\omega_{D}$ is the dipole energy, $\varepsilon_{0}$ is the vacuum permittivity, and *V*_gap_ represents the gap volume (fig. S3, E and F) (*28*). As the gap narrows (Fig. 2K), the field strength increases and surpasses the 10 V/nm threshold (*31*). However, extrapolating this trend to sub–0.2 nm gaps is not meaningful because 0.2 nm (~2 Å) is on the order of atomic dimensions [half the Au lattice constant (*32*)], where continuum electrodynamics breaks down and tunneling/atomistic effects dominate.

To quantify fields in subnanometer gaps, we computed the electron beam–induced fields by the CEM and QCM (Fig. 2K). Unlike the rough analytical estimate, both predict a pronounced and nonlinear dependence on gap size. When gap conductivity is incorporated, the QCM indicates that the field-induced tunneling threshold is reached for gaps ≲0.6 nm (6 Å).

### Electron beam–induced electric field

**Fig. 3.**
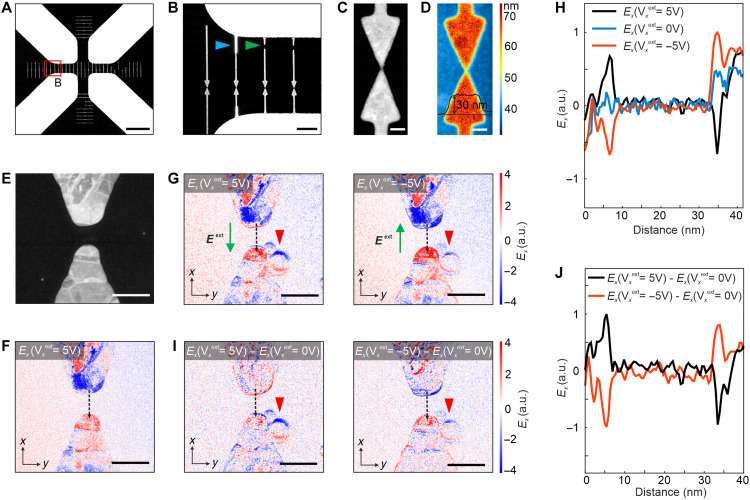
Visualizing electrical polarization switching. (**A**) SEM image of the nanostructures fabricated on the SiN*_x_* membrane attached to the electrical biasing chip. The small features on the SiN*_x_* membrane are the nanofabricated Au bowties connected to the large electrodes. (**B**) HAADF-STEM image of the area marked in the red frame in (A). The areas marked by the green and blue arrows show the bowties disconnected by the Ga^+^ ion beam and connected by the ion beam deposition of platinum, respectively. (**C** and **D**) HAADF-STEM image of a bowtie and its corresponding thickness map. (**E**) Close-up HAADF-STEM image of the hotspot in a Au bowtie. (**F**) 4D-STEM electric field map along the *x* axis (*E*_x_) for the bowtie shown in (E). (**G**) *E_x_* maps obtained at the electrical biasing values of 5 and −5 V. The direction of the applied field is indicated by the green arrows in (G). (**H**) Field profiles recorded along the dashed black lines in (F) and (G). (**I**) 4D-STEM electric field maps obtained by subtracting the field map recorded at 0 V from the *E_x_* maps recorded at 5 and −5 V. The red triangles mark the position of electron beam–induced contamination in (G) and (I). (**J**) Field profiles recorded along the dashed black lines in (I). Scale bars: 10 μm (A), 1 μm (B), 100 nm [(C) and (D)], and 50 nm [(E) to (G) and (I)].

To characterize the electron beam–induced electric field distribution and quantify the field strength at emitter sites, we perform 4D-STEM experiments in conjunction with FEM simulations (section S1). Unlike EEL spectroscopy (EELS), which can only detect the *z*-component of the induced electric field, 4D-STEM enables the mapping of lateral electric field components (*33*–*38*). However, the field distribution observed in 4D-STEM field maps is influenced by two primary mechanisms that cannot be disentangled: (i) Edge effect: The potential difference between Au-T and SiN*_x_* induces localized regions of high field intensity, especially at the edge and apex of Au-T (*39*–*41*). (ii) LSPR excitation: The incident electron can excite LSPRs on Au-T and subsequently undergo deflection because of the electric field generated by charge polarization (*33*). At a beam current of 25 pA in STEM mode, the electron arrival time at Au-T is 6.41 ns. Given the ultrashort lifetime of LSPRs (5.34 fs for dipolar LSPRs) (*42*), the deflection of subsequent electrons by evanescent plasmonic fields is negligible. Position-averaged convergent beam electron diffraction (PACBED) is an effective diagnostic for crystal thickness and orientation artifacts in 4D-STEM field mapping (*43*). Strong patterns obtained from the interior of Au-T indicate pronounced dynamical scattering, while weak patterns at edges reflect geometric averaging and reduced thickness, not the absence of edge fields (fig. S9). The apparent weak edge effect in PACBED is thus expected and consistent with the edge-localized fields observed in 4D-STEM field maps. Given that SiN*_x_* support is amorphous and thin, PACBED reduces to a featureless and centered disk with no crystalline modulations (fig. S9B). Despite the inherent challenges in 4D-STEM field mapping, the electric field associated with charge polarization can be distinguished from the edge effect by analyzing the response to an externally applied field between two closely spaced Au-T tips (Fig. 3).

To visualize electrical polarization switching under an external electrical field, we prepare a sample of 30-nm-thick Au nanostructures on a SiN*_x_* membrane (Fig. 3, A to D). After fabrication, we introduce small gaps between adjacent Au-Ts using a focused electron beam by STEM. This approach enables removing the junction between two triangles and creating a 7-nm gap (fig. S10, A to C). To ensure accurate electrical measurements, we clean all samples with O_2_ plasma before the electrical measurements (fig. S11). We perform 4D-STEM electric field (*E_x_*) mapping under both unbiased and biased conditions. Without an external bias, the field maps show charge polarization at the triangle apexes (Fig. 3, E and F). When we apply +5-V and −5-V biases, the charge polarization shifts distinctly, particularly at the sharp triangle tips (Fig. 3, G and H). This shift becomes more pronounced when we subtract the unbiased field map from those obtained under bias (Fig. 3, I and J). Imaging before and after bias confirms that voltages between −5 V and +5 V do not alter the morphology of the nanostructures (fig. S12). When we apply a lateral bias across two tips, 4D-STEM reveals strong transverse fields at the edges, where the projected potential varies rapidly. In contrast, the gap center shows little or no signal because the measurable transverse field remains weak. In summary, 4D-STEM electric field mapping shows that charges accumulate at the apexes of the Au-T tips and that applying bias effectively switches electrical polarization (Fig. 3).

**Fig. 4.**
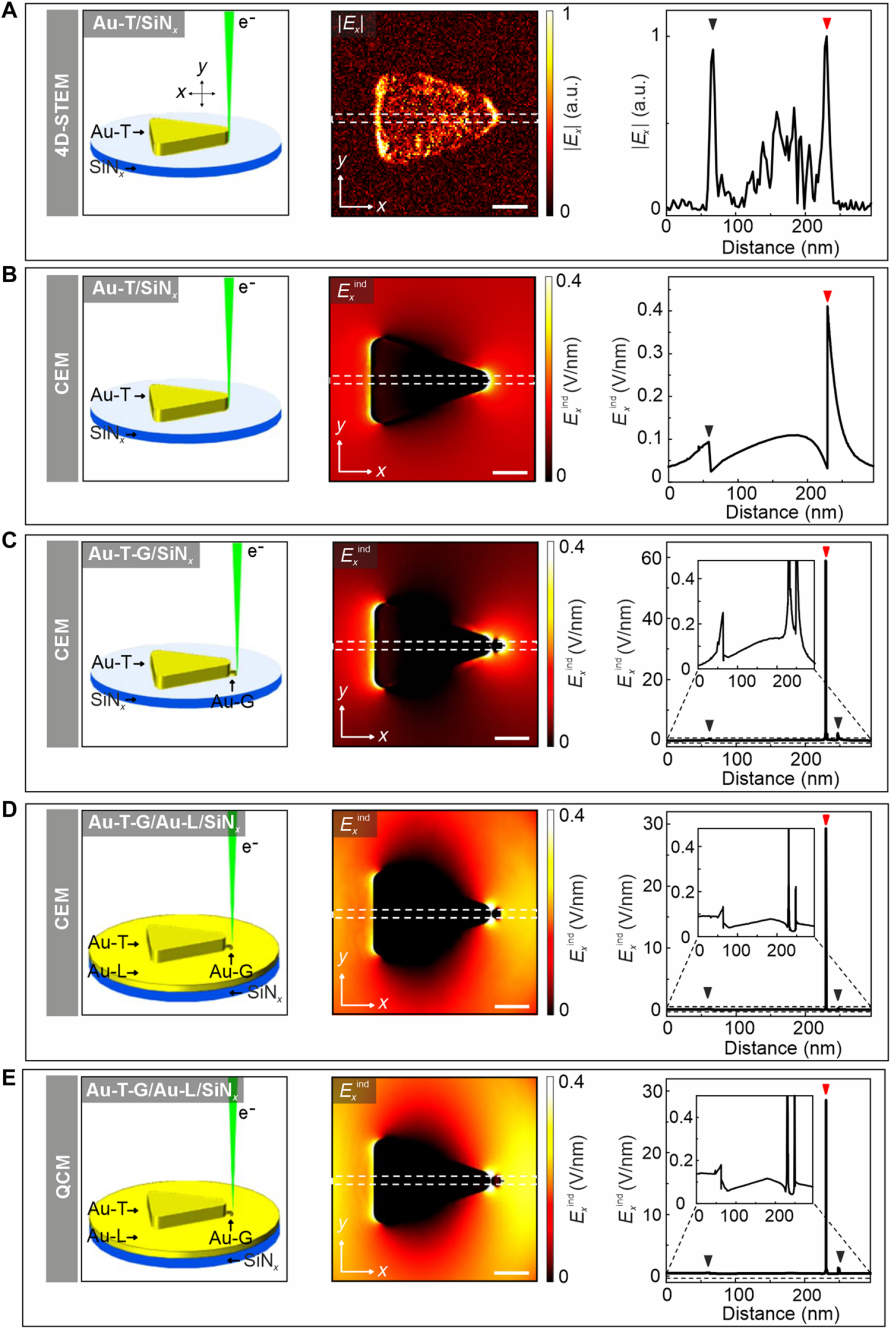
Electron beam–induced electric field. (**A**) Schematic representation of the Au-T-G/SiN*_x_* structure used in 4D-STEM measurements, corresponding 4D-STEM electric field map (|*E_x_*|) obtained at 2 V, and field profile extracted along the white dashed frame in the field map. (**B**) Schematic of the Au-T/SiN*_x_* model used in the CEM, computed electron beam–induced electric field (*E*^ind^) map at the dipole energy, and corresponding electric field strength profile recorded along the white frame in the field map. (**C**) Schematic of the Au-T-G/SiN*_x_* model used in the CEM, computed *E*^ind^ map at the dipole energy, and field strength profile extracted along the white frame. (**D**) Schematic of the Au-T-G/Au-L/SiN*_x_* model used in the CEM, computed *E*^ind^ map at the dipole energy, and corresponding field strength profile recorded along the white frame. (**E**) Schematic of the Au-T-G/Au-L/SiN*_x_* model used in the QCM, computed *E*^ind^ map at the dipole energy, and corresponding field strength profile recorded along the white frame. The *R*_tip_ is 15 nm for all structures shown in (A) to (E), and the separation between the Au-T tip and Au-G is 0.5 nm in the simulated structures presented in (C) to (E). The blue and red triangles shown on electric field profiles in (A) to (E) mark the locations of edge and tip at Au-Ts. Scale bars: 50 nm [(A) to (E)].

To provide further insight into the observed field distribution, we examine simulated field maps on the basis of a Au-T model that mimics the experimental structure. The field distribution observed in the 4D-STEM electric field maps closely matches the *x*-component of the calculated induced electric field (*E_x_*^ind^) for a comparable Au-T/SiN*_x_* configuration (Fig. 4, A and B). The simulated field maps confirm that the field intensity is substantially higher at the tip than at the edges of the Au-T. The amplitude of the induced field, given by *E_x_*^ind^ = *gE*_0_, where *E*_0_ is the background electric field, is calculated to be 0.41 V/nm at the Au-T tip, yielding a field enhancement factor of *g* ≈ 21.

To further investigate field enhancement effects, we conduct electromagnetic simulations with the CEM for a Au-T-G/SiN*_x_* structure (Fig. 4C). Similar to the isolated Au-T, a strong localized field is observed at the edges and tip of the Au-T-G emitter. In this coupled configuration, *E_x_*^ind^ reaches 58.86 V/nm (*g* ≈ 2943), representing a substantial increase compared to the standalone Au-T/SiN*_x_*. For the Au-T-G/Au-L/SiN*_x_* system, the induced electric field is slightly reduced to *E_x_*^ind^ = 29.28 V/nm (*g* ≈ 1464). However, it remains substantially higher than that of Au-T/SiN*_x_* (Fig. 4D). Although the presence of the Au-L beneath the Au-T-G reduces the induced field compared to the Au-T-G/SiN*_x_* configuration, the coupling between the Au-T and the Au-G still generates a strong field exceeding 10 V/nm. The QCM applied to the Au-T-G/Au-L/SiN*_x_* system yields a comparable result but with a slightly reduced induced field, *E_x_*^ind^ = 28.16 V/nm (*g* ≈ 1408).

The *E_x_*^ind^ computed by the QCM indicates that the Au-T-G/Au-L/SiN*_x_* system exhibits a field enhancement, which is ~69 times greater than that of the Au-T/SiN*_x_*, primarily due to plasmonic coupling that amplifies local field intensities. In contrast, a Au-G/SiN*_x_* structure alone produces a weaker induced field of ~0.2 V/nm (*g* ≈ 10; fig. S13). Additional FEM simulations suggest that electron emission efficiency could be further enhanced by increasing the number of Au-Gs surrounding the Au-T tips (fig. S14).

### Giant field enhancement confirmed by optical excitations

**Fig. 5.**
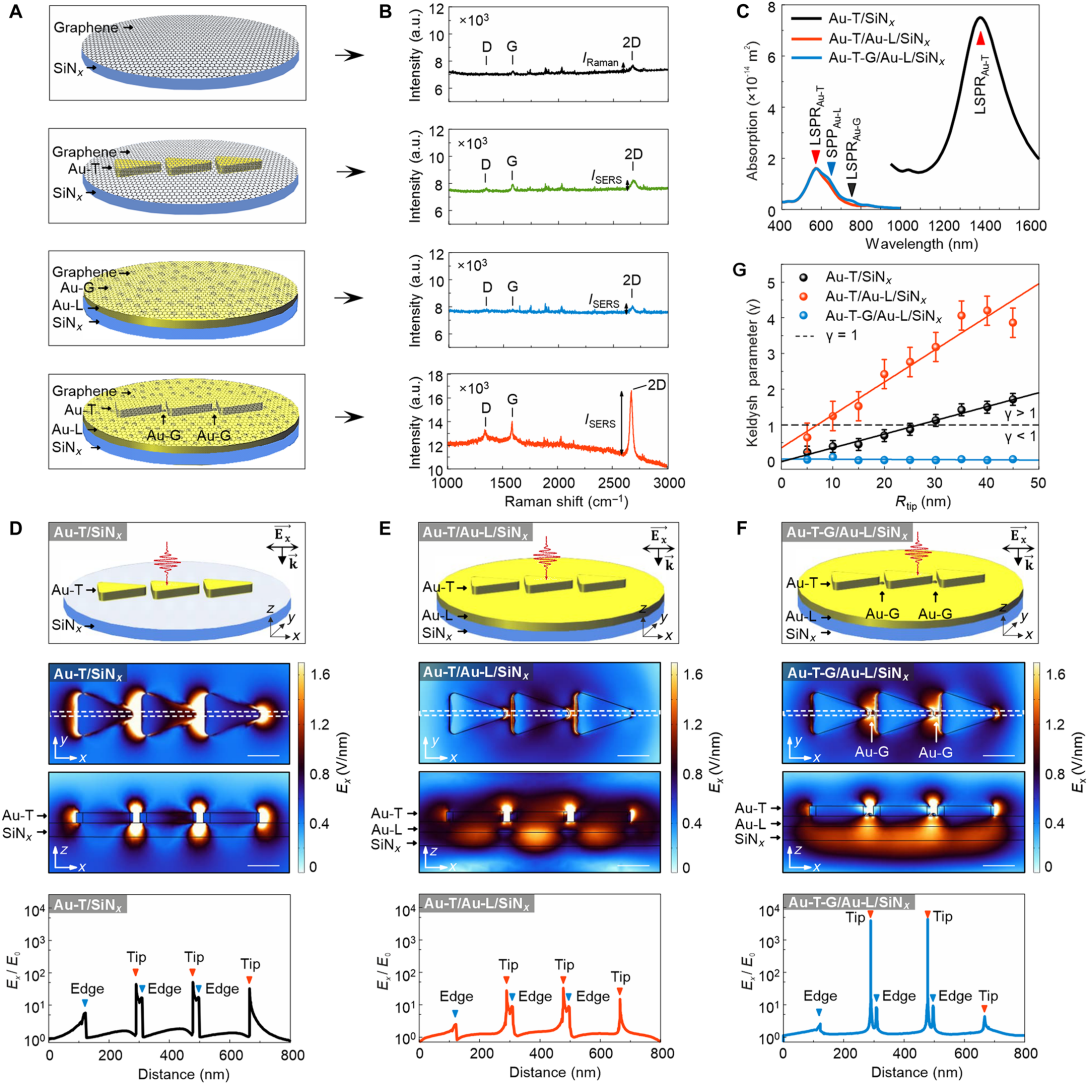
Optical field enhancement. (**A**) Schematic representation of the monolayer graphene–coated structures used in SERS measurements, including SiN*_x_*, Au-T/SiN*_x_*, Au-L/SiN*_x_*, and Au-T-G/Au-L/SiN*_x_*. (**B**) Raman spectra of monolayer graphene deposited on the substrates illustrated in (A), highlighting variations in SERS enhancement. (**C**) Computed absorption spectra for Au-T/SiN*_x_*, Au-T/Au-L/SiN*_x_*, and Au-T-G/Au-L/SiN*_x_*, illustrating plasmonic resonances and their spectral shifts resulting from structural coupling effects. (**D**) Schematic of the Au-T/SiN*_x_* structure used in FEM simulations, corresponding computed electric field distribution along the *x* axis, and field enhancement profile extracted along the dashed white line in the field map. (**E**) Schematic of the Au-T/Au-L/SiN*_x_* structure used in FEM simulations, along with its computed electric field distribution along the *x* axis and corresponding field enhancement profile. (**F**) Schematic of the Au-T-G/Au-L/SiN*_x_* structure used in FEM simulations, its computed electric field distribution along the *x* axis, and associated field enhancement profile. In this structure, the gap between the Au-T tip and Au-G is set to 0.5 nm. The electric field distributions presented in (D) to (F) are computed at the dipolar LSPR energies derived from the absorption spectra in (C). The blue and red triangles shown on electric field profiles in (D) to (F) mark the locations of edge and tip at Au-Ts. (**G**) Keldysh parameter (γ) calculated for the structures shown in (D) to (F) as a function of *R*_tip_ varying from 5 nm to 45 nm in FEM simulations. The error bars represent the standard error of the mean. Scale bars: 100 nm [(D) to (F)].

Next, we conduct SERS measurements on monolayer graphene–coated structures to experimentally verify the extreme field enhancement at the apex of the emitter tips. In general, the SERS enhancement factor, which depends on the squared amplitude of both the excitation and scattered fields, is given by (*E*^ind^/*E*_0_)^4^, where *E*^ind^ = *gE*_0_ represents the amplitude of the locally induced field at the Raman-active site, and *E*_0_ is the incident field amplitude (section S2 and fig. S15). Consistent with a previous study (*44*), the enhancement factors are calculated as ~15 and 17 for graphene-coated Au-T/SiN*_x_* (*g* ≈ 2.02) and Au-L/SiN*_x_* (*g* ≈ 1.96), respectively (Fig. 5, A and B). However, for graphene-coated Au-T-G/Au-L/SiN*_x_*, the enhancement factor increases substantially to ~10^8^, comparable to values reported for hexagonal boron nitride–coated Au nanoparticles coupled to a Au film (*45*). Consistent with the computational results presented in the previous section, SERS measurements reveal that the induced field in Au-T-G/Au-L/SiN*_x_* (*g* ≈ 147) is ~73 times stronger than that in Au-T/SiN*_x_* (*g* ≈ 2.02). To corroborate the enhancement, we also measured SERS on a control sample containing only Au grains (fig. S16). Under 488-nm excitation, the Au-T-G/Au-L/SiN*_x_* structure shows an enhancement factor of *g* ~ 143.8, which is ~76 times higher than that of Au-T/SiN*_x_* (*g* ~ 1.88). These results are consistent with our previous measurements.

To further elucidate the underlying mechanism of this field enhancement, we perform FEM simulations incorporating optical excitations (section S3). Consistent with the results in Fig. 2 (A, D, and F), the dipolar LSPR of Au-T exhibits a blue shift when placed on the Au-L, approaching the surface plasmon polariton (SPP) energy of the Au-L substrate (Fig. 5C). The dipolar LSPR of Au-T/SiN*_x_* appears at 0.88 eV (1400 nm) because of near-field coupling among closely spaced Au-Ts, whereas the dipolar LSPR of an isolated Au-T on SiN*_x_* is positioned at 1.1 eV (Fig. 2A and fig. S17).

Analysis of the *x*-component of the electric field distribution confirms that field confinement is notably stronger in Au-T-G/Au-L/SiN*_x_* than in Au-T/SiN*_x_* or Au-T/Au-L/SiN*_x_* (Fig. 5, D to F). In addition, in the Au-T-G/Au-L/SiN*_x_* system, the field penetrates beneath the emitters into the underlying Au-L, whereas no substantial field penetration is observed beneath emitters on SiN*_x_* (Fig. 5, D to F). These findings indicate that Au-T-G/Au-L/SiN*_x_* (*g* ≈ 4359) exhibits a field enhancement ~99 times greater than Au-T/SiN*_x_* (*g* ≈ 44). In contrast, the enhancement factor for Au-T/Au-L/SiN*_x_* (*g* ≈ 32) is slightly lower than that for Au-T/SiN*_x_*, while Au-G/Au-L/SiN*_x_* (*g* ≈ 6.6) shows the weakest enhancement, which is consistent with the simulated electron beam–induced field distributions (figs. S13 and S18).

Furthermore, we investigate the dependence of field emission on the *R*_tip_ and *g* (Fig. 5G). For Au-T/SiN*_x_*, field emission occurs when *R*_tip_ < 30 nm, where γ < 1 [*W*_F_ = 4.9 eV for Au (*46*)]. In the case of Au-T/Au-L/SiN*_x_*, field emission dominates when *R*_tip_ < 10 nm (γ < 1), while multiphoton photoemission emerges for *R*_tip_ > 10 nm (γ > 1). When Au-Ts are not coupled, field emission is restricted to *R*_tip_ < 5 nm (γ < 1), as shown in fig. S19. Instead, the Au-T-G/Au-L/SiN*_x_* system exhibits a unique behavior in which γ remains independent of *R*_*t*ip_ with γ < 1 for all tip radii as a result of strong field localization at the emitter hotspot (Fig. 5G).

### In situ detection of field emission in the TEM

To experimentally measure field emission resulting from the strong electric fields at emitter hotspots, we conduct in situ electrical measurements on electron beam–irradiated emitters within the TEM (Fig. 1A). The steady-state current balance in the TEM is described by *I*_B_ = *I*_T_ + *I*_SE_ + *I*_BS_ + *I*_A_ + *V*_s_/*R*_s_, where *I*_B_ is the incident electron-beam current, *I*_T_ represents the transmitted beam current, *I*_SE_ is the SE current, *I*_BS_ is the current as a result of backscattered electrons, *I*_A_ is the absorbed beam current, and *V*_s_ and *R*_s_ are the sample potential and effective resistance to ground, respectively (*47*). Given that *I*_T_ ~ *I*_B_, *I*_A_, and *I*_BS_ are negligible (see section S4) in the TEM setup, the steady-state current balance simplifies to *I*_B_ = *I*_T_ + *I*_SE_ + *V*_s_/*R*_s_. In our experiments, we measure the field emission current (*I*_FE_) from electron beam–excited Au-T-G emitters along with *I*_SE_. The total measured current is then given by *I*_total_ = *I*_FE_ + *I*_SE_ + *I*_BC_, where *I*_BC_ is the background current. Here, the current is detected under parallel-beam illumination of the 4584 Au-T-G emitters within the 15 μm–by–15 μm field. Given typical STEM pixel dwell times in the microsecond range and plasmon lifetimes in the femtosecond range, the emission signal from a single emitter under focused-beam irradiation is below our detection limit.

**Fig. 6.**
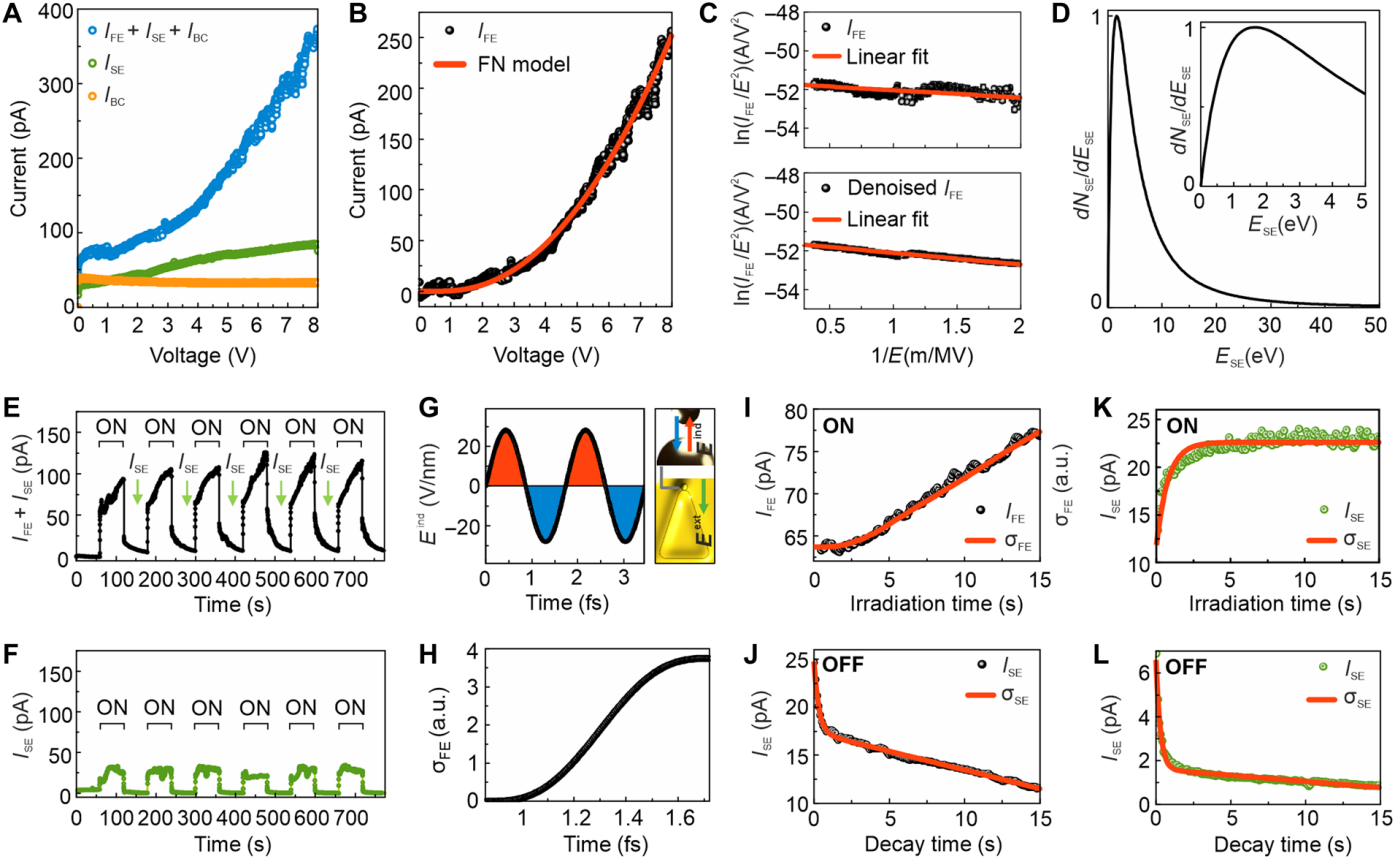
In situ electrical measurements in the TEM. (**A**) Current-voltage characteristics of Au-T-G/Au-L/SiN*_x_* (blue data points) and Au-G/Au-L/SiN*_x_* (green data points) under parallel electron-beam irradiation, as well as Au-T/Au-L/SiN*_x_* (orange data points) in the absence of electron-beam exposure. The *E*^ext^ driving electrons from the emitter to the collector is varied from 0 to 2.7 V/μm. (**B**) Experimentally measured field emission current as a function of applied bias voltage. The data reveal FN emission after subtracting contributions from SEs and parasitic capacitance. The red line represents the FN model fit. (**C**) FN plot derived from both raw and denoised field emission current (*I*_FE_). (**D**) Normalized energy distribution of SEs. (**E** and **F**) Time-dependent response of Au-T-G/Au-L/SiN*_x_* and Au-G/Au-L/SiN*_x_* devices operated at a bias voltage of 4 V, respectively. (**G**) Two cycles of oscillating *E*^ind^(*t*), which is used for calculating $\sigma_{\text{FE}}$. The field emission occurring in the blue-shaded region is directed along the *x* axis of the emitter. The blue and red arrows indicate *E*^ind^ oriented in the negative and positive *x* directions, respectively, while the green arrow represents *E*^ext^ directed in the negative *x* direction. (**H**) Computed $\sigma_{\text{FE}}$ for a half-cycle of the oscillating field corresponding to the blue-shaded region in (G). (**I**) Time evolution of *I*_FE_ and corresponding computed $\sigma_{\text{FE}}$ as a function of irradiation time for Au-T-G/Au-L/SiN*_x_*. (**J**) Time evolution of *I*_SE_ and computed $\sigma_{\text{FE}}$ following electron-beam turn-off for Au-T-G/Au-L/SiN*_x_*. (**K**) Time evolution of *I*_SE_ and computed $\sigma_{\text{SE}}$ as a function of irradiation time for Au-G/Au-L/SiN*_x_*. (**L**) Time evolution of *I*_SE_ and computed $\sigma_{\text{FE}}$ after electron-beam turn-off for Au-L/SiN*_x_*.

Figure 6A shows the bias dependence of *I*_total_, *I*_SE_, and *I*_BC_, which are measured at specific beam positions and device configurations (fig. S20). By subtracting *I*_SE_ and *I*_BC_, we isolate *I*_FE_ (Fig. 6B), which is found to closely match the FN field emission model given in Eq. 1

$$I_{\text{FE}}=a{\left(E^{\text{ind}}\right)}^{2}\text{exp}\left(-b/E^{\text{ind}}\right)$$ (1)

where *a* and *b* are constants (*24*). The induced electric field *E_x_*^ind^ is calculated to be 28.16 V/nm by the QCM for emitters with a gap distance of 0.5 nm. Considering variations in the gap spacing for individual emitters, we estimate *E*^ind^ = 29.93 V/nm using SERS, revealing that *E*^ind^ on Au-T-G/Au-L/SiN*_x_* is 73 times stronger than that on Au-T/SiN*_x_*, producing *E*^ind^ = 0.41 V/nm. Given that γ = 0.90 for *E*^ind^ = 29.93 V/nm (where γ < 1), tunneling ionization is favored. The maximum *E*^ext^ driving the emitted electrons toward the collector reaches 2.7 V/μm, which is insufficient to initiate field emission. Consequently, FN tunneling in our experiments is primarily induced by the plasmon-enhanced localized near-fields. This strong-field tunneling emission is further validated by the FN plot (Fig. 6C and fig. S21).

The SE energy distribution for Au is described by Eq. 2

$$dN_{\text{SE}}/dE_{\text{SE}}=\left(1/E_{B}\right)\left[E_{\text{SE}}/{\left(E_{\text{SE}}+W_{F-\text{Au}}\right)}^{4}\right]$$ (2)

where *E*_B_ is the primary electron-beam energy, and *E*_SE_ is the energy of SEs relative to the vacuum level (*48*, *49*). SEs are emitted over a broad energy range (<50 eV), with a peak at 1.6 eV, which is below the *W*_F_ (Fig. 4D). Instead, field emission is an exponentially driven process that scales with the local electric field strength. While stronger fields increase the probability of SE escape, they do not directly enhance SE generation that depends on the incident beam energy and inelastic scattering processes (Fig. 6A).

Figure 6 (E and F) illustrates the time dependence of *I*_FE_ and *I*_SE_ for electron-beam on/off cycles in Au-T-G/Au-L/SiN*_x_* and Au-G/Au-L/SiN*_x_*. Here, the background current *I*_BC_ is subtracted from both *I*_total_ and *I*_SE_. Figure 6E demonstrates that the current increases instantaneously when the beam is turned on and decays exponentially when the beam is switched off. When the beam is on, *I*_FE_ emitted from Au-T-G emitters includes contributions from *I*_SE_, which persists even after turning off the primary beam because of trapped charges. For Au-G/Au-L/SiN*_x_*, only *I*_SE_ is detected when the beam is on (Fig. 6F), and unlike Au-T-G/Au-L/SiN*_x_*, *I*_SE_ drops much more rapidly once the beam is off.

The total field emission yield $\sigma_{\text{FE}}$ given in Eq. 3 (*9*, *24*) is computed by integrating the partial currents generated by the induced electric field waveform *E*^ind^(*t*) at the emitter (Fig. 6G).

$$\sigma_{\text{FE}}\left(t\right)=f_{R}\times \int_{-T_{R}/2}^{T_{R}/2}a^{*}{\left[E^{\text{ind}}\left(t\right)\right]}^{2}\text{exp}\left[-\frac{b^{*}}{E^{\text{ind}}\left(t\right)}\right]\mathrm{dt}$$ (3)

where *T*_R_ = 1/*f*_R_. Field emission directed along the *x* axis arises from the negative half-cycles of the induced field oscillating at the emitter’s plasmon frequency (Fig. 6H). Given that *E*^ind^ ≫ *E*^ext^, electrons emitted from Au-Gs toward Au-Ts cannot be detected by the collector. In Fig. 6I, the measured *I*_FE_ increases with irradiation time, and the $\sigma_{\text{FE}}$ integrated over 15 s shows a similar trend. At the emitter hotspots, where the fields are strong, SEs with energies below *W*_F_ may also contribute to FN emission. To explore the dynamics of SEs during beam on/off cycles, we define $\sigma_{\text{SE}}=I_{\text{SE}}/I_{B}$ (*50*). The time dependence of $\sigma_{\text{SE}}\left(t\right)$ is given by Eq. 4

$$\sigma_{\text{SE}}\left(t\right)={\sigma_{\text{SE}}}^{\infty }+\left({\sigma_{\text{SE}}}^{0}-{\sigma_{\text{SE}}}^{\infty }\right)\text{exp}\left(-t/\tau \right)$$ (4)

where ${\sigma_{\text{SE}}}^{0}$ and ${\sigma_{\text{SE}}}^{\infty }$ are the initial and steady-state values, respectively; τ is the time constant; and *t* is the irradiation time (*51*). Trapped SEs can release their energy over extended timescales that enable the delayed excitation of subsequent SEs even after the primary beam is switched off. When the beam is turned off, the field emission vanishes and the current rapidly drops to ~25 pA because of continued SE emission before decaying exponentially (Fig. 6J). Both *I*_SE_ and $\sigma_{\text{SE}}$ exhibit an exponential decay over time because of charge recombination and detraining mechanisms that reduce the density of trapped electrons after the beam is switched off (*51*).

Unlike field emission, SE emission recorded over 15 s from Au-G/Au-L/SiN*_x_* reaches saturation within 3 s of irradiation (Fig. 6K), while the excitation energy remains constant during the measurement. Upon turning off the beam, *I*_SE_ initially drops sharply to ~6 pA, followed by an exponential decay (Fig. 6L). This behavior is consistent with the data shown in Fig. 6J. However, *I*_SE_ recorded on Au-G/Au-L/SiN*_x_* is substantially lower than that observed on Au-T-G/Au-L/SiN*_x_*. The higher *I*_SE_ observed from Au-T-G/Au-L/SiN*_x_* is attributed to the efficient plasmon energy transfer to SEs excited at the emitter hotspots (section S5). We find that the energy transfer from LSPRs to SEs excited at the emitter hotspots is more efficient than the transfer from SPPs to SEs in Au-G/Au-L/SiN*_x_*. Although hot electrons generated by plasmon decay can also excite SEs, this mechanism is less effective for Au, given that *W*_F_ is higher than the plasmon energy (section S6) (*52*). Overall, field emission predominates over SE emission because of the large field enhancement at the emitter hotspots where tunneling occurs.

Hot-electron emission is not considered in this context because the hot electrons generated in Au cannot escape into the vacuum because of its relatively high work function compared to its plasmon energies (section S7) (*53*). Furthermore, thermal emission is excluded, given that Au exhibits efficient heat conduction and the volume being heated is relatively small. Using Richardson’s law (*48*), we estimate that the highest current observed in Fig. 6B corresponds to a temperature of 1545 K, which cannot be solely attributed to plasmonic heating as it exceeds the melting point of Au.

## DISCUSSION

This study presents a coherent picture of how plasmon-enhanced near-fields enable efficient tunneling field emission at the hotspots of coupled plasmonic nanostructures under intense electron-beam irradiation. Our experimental results demonstrate that the emitter structures, comprising Au-Ts coupled to hemispherical Au-Gs on a conductive substrate, can localize and amplify electric fields to magnitudes exceeding 10 V/nm, which is sufficient to surpass the threshold for FN tunneling. Crucially, the recorded emission currents exhibit clear FN characteristics, unambiguously confirming the tunneling origin of the observed electron emission and distinguishing them from SE contributions.

These findings have broad implications across multiple research domains. In the context of electron microscopy and ultrafast electron dynamics, the ability to generate field emission from well-defined nanoscale emitters under electron irradiation opens additional possibilities for realizing spatially confined and temporally switchable electron sources. The integration of these emitter structures with ultrafast detectors or on-chip photonic circuitry offers notable potential for the development of compact devices that are capable of high-speed signal generation, dynamic light modulation, and electron beam–based sensing. The proposed emitter structures could also be integrated into emerging technologies including attosecond electron microscopes (*12*) and compact free-electron light sources. Moreover, the correlation between strong SERS activity and electron beam–induced field emission at the same sites indicates that these sites can be co-optimized for multifunctional plasmonic systems, enabling both sensing and emission for applications in quantum plasmonics, nanoscale optoelectronics, and hybrid photonic-electronic devices.

By combining 4D-STEM with in situ electrical measurements in the TEM, we achieve spatially resolved mapping of the lateral components of electric fields and direct visualization of electrical polarization switching in lithographically defined plasmonic nanostructures. 4D-STEM provides nanometer-scale maps of the projected electric field. While prior studies have focused on isolated or planar structures (*35*, *37*, *38*, *43*, *54*), here, we apply the method to coupled plasmonic nanoparticles with subnanometer gaps, where near-field interactions generate strong and spatially varying lateral field components that can be quantified with nanometer precision. Notably, the detection of electrically driven polarization switching between adjacent tips of the nanostructures provides critical insights into charge redistribution dynamics and field localization mechanisms. However, the interpretation of 4D-STEM field maps remains challenged by pronounced edge effects, which can dominate the fields induced by LSPR excitation. Despite these limitations, the technique allows for direct real-space visualization of polarization switching processes in electrically biased metallic nanostructures. In addition, the present experiments, performed under continuous electron-beam illumination, do not capture the ultrafast dynamics associated with transient charge oscillations or femtosecond-to-attosecond tunneling events. Future time-resolved studies, particularly using attosecond electron pulses, will be essential to directly observe transient charge oscillations and tunneling events at the emitter sites.

In summary, we have demonstrated in situ measurements of electron beam–driven field emission from plasmonic nanostructures irradiated by a 200-kV electron beam within a TEM. The fabricated electron emitters not only exhibit pronounced field emission but also display notable surface-enhanced Raman scattering activity, attributed to the strong localized electric fields at emitter hotspots. The spatial distribution of the electron beam–induced field and the electrical polarization switching are visualized by 4D-STEM. Computational and experimental field maps show that the highest peak field intensity occurs at the apex of the emitter tips, where the field enhancement is maximized. In situ TEM electrical measurements provide direct evidence for FN field emission, which is substantially stronger than SE emission under the same experimental conditions. These findings establish a platform for probing ultrafast field emission dynamics at the nanoscale, with the potential to advance electron microscopy, miniaturized particle accelerators, sensor technologies, displays, and optical communication systems. Building on prior demonstrations of 4D-STEM electric field mapping under in situ electrical biasing (*38*), we apply this approach to lithographically defined plasmonic nanoparticles to visualize spatially resolved field maps and electrical polarization switching.

## MATERIALS AND METHODS

### Nanofabrication

The emitter arrays and electrode structures were fabricated directly on in situ TEM biasing chips with SiN*_x_* membranes, referred to as E-chips (Protochips Inc.) using electron-beam lithography. Before spin-coating with polymethyl methacrylate (PMMA), the E-chips were cleaned via oxygen plasma treatment under the following conditions: 40% O_2_ and 60% Ar with a total gas flow of 30 standard cubic centimeter per minute for 3 min at 95% power. Subsequently, an 80- to 90-nm-thick layer of PMMA (2.5% PMMA 950k in anisole) was spin-coated at 6000 rpm (acceleration rate: 2000 rpm/s) for 35 s, followed by annealing at 160°C for 4 min. Resist patterning was performed using a Raith eLine electron-beam lithography system equipped with a 7.5-μm objective aperture and operated at an acceleration voltage of 15 kV. The working distance, beam current, and areal dose were set at 9 mm, 15.2 pA, and 1300 μC/cm^2^, respectively. Following exposure, the resist was developed in a methyl isobutyl ketone/isopropyl alcohol solution (3:1) at 0°C for 30 s and then dried using a nitrogen spray gun. After resist development, a 30-nm-thick layer of Au (99.99% purity) was deposited via thermal evaporation (Univex 1) at a base pressure of ~3 × 10^−7^ mbar. The deposition rate, monitored using a quartz crystal microbalance, was ~1.4 Å/s. Metal liftoff was carried out by immersing the sample in *N*-methyl-2-pyrrolidone heated to 60°C for ~30 min, followed by sequential rinsing with acetone and isopropanol. Using this fabrication process, Au electrodes (15 μm by 15 μm) were first patterned on SiN*_x_* membranes supported by E-chips, and emitter arrays were subsequently fabricated on select electrode regions (fig. S1). Before electrical measurements in the TEM, E-chips containing emitter arrays were subjected to oxygen plasma cleaning. Here, the emitter arrays including 30-nm-thick elongated Au-Ts are on prepatterned 30-nm-thick Au-Ls, which are deposited onto 40-nm-thick SiN*_x_* membranes on E-chips.

### STEM and TEM imaging

STEM and TEM imaging of the emitters on E-chips was performed using a JEOL ARM200F FEG-STEM/TEM. This system is equipped with a cold field-emission gun, a postspecimen spherical aberration corrector (Cs), and a Gatan GIF Quantum ERS EEL spectrometer. Imaging was conducted at an acceleration voltage of 200 kV under underfocus conditions.

### In situ electrical measurements in the TEM

Electrical biasing experiments were performed using a Protochips Aduro single-tilt eight-pin holder and a Protochips Fusion double-tilt six-pin holder (Protochips Inc.), controlled via a Keithley 2636B System SourceMeter. The measurement protocol followed a three-step waveform mode: (i) a linear increase in current over 300 s, (ii) a constant current phase for 60 s, and (iii) a linear decrease over 300 s. The time dependence of the emission current was recorded by alternately switching the electron beam on for 60 s and off for 60 s while maintaining a constant bias voltage. All in situ electrical biasing experiments were conducted in a JEOL ARM200F FEG-STEM/TEM operated at 200 kV.

### Focused ion beam (FIB) process

A focused ion beam (FIB) system (FEI Scios DualBeam, Thermo Fisher Scientific Inc.) equipped with a Ga^+^ ion source was used to create and refine electrical contacts between the prepatterned electrodes on the E-chip. The FIB system was operated at an acceleration voltage of 30 kV, with beam currents of either 1.5 or 10 pA, depending on the specific processing requirements.

### Electron-beam manipulation

The nanogaps in connected Au-Ts, used for the detection of electrical polarization switching via 4D-STEM, were created through focused electron-beam manipulation at their junctions. This was performed using a JEOL ARM200F STEM/TEM with a probe Cs corrector. A 200-kV electron beam was directed at the nanostructures for ~1 min, inducing knock-on damage that enabled controlled formation of nanoscale gaps. To achieve precise separation, the beam was focused on various positions on junctions. A probe size of 2C, with a beam diameter of 0.27 nm, was chosen to ensure high current density. Experiments were conducted at a beam current of 15 μA and a current density of 83 pA/cm^2^.

### Electron dose measurements

The electron dose on the emitters was quantified using the AXON Dose system (Protochips Inc.), which includes a TEM calibration holder with an integrated Faraday cup at the tip position, along with the AXON Dose calibration software module (*55*). The measured beam current was 8872 pA over a 15 μm–by–15 μm area when the TEM was operated at a 200-kV acceleration voltage. The beam current measured in STEM mode was 25 pA at the same acceleration voltage.

### Electromagnetic simulations

Classical electromagnetic simulations were performed using the FEM implemented in the commercial solver COMSOL Multiphysics to compute the electron beam–induced electric field. The optical properties of Au were incorporated using linear interpolation, while the refractive index of SiN*_x_* was set to 2.4, as its dispersion was negligible within the spectral range of interest.

EELS simulations were conducted over an energy range of 0.3 to 3 eV with an energy step of 0.01 eV. Maxwell’s equations were solved in the frequency domain using the MUMPS (multifrontal massively parallel sparse) direct solver. In the FEM model, the electron beam was represented as a line current with a spectral current density given by Eq. 5

$$j\left(z,\omega \right)=-e\hat{z}\delta \left[R-R_{0}\right]e^{i\omega z/v}$$ (5)

where $R_{0}=\left(x_{0},y_{0}\right)$ is the impact parameter of the electron trajectory, and ω is the angular frequency.

The plasmonic nanostructures and electron-beam interactions were simulated within a spherical perfectly matched layer (PML) to absorb scattered electromagnetic waves. The induced electric field was obtained by first computing the background electric field $E_{0}\left(r,t\right)$ for an empty PML and then determining the total field E(r,ω) when the plasmonic nanostructure was introduced. The induced electric field was extracted as Eq. 6

$$E^{\text{ind}}\left(r,\omega \right)=E\left(r,\omega \right)-E_{0}\left(r,\omega \right)$$ (6)

The dielectric function for Au was taken from Johnson and Christy (*56*). Additional simulation details are provided in section S1.

The energy loss probability $\Gamma_{\text{EELS}}\left(\omega \right)$ was extracted from the induced electric field along the source current, following Eq. 7

$$\Gamma_{\text{EELS}}\left(\omega \right)=\frac{\mathrm{ve}}{2\pi ћ\omega }\int \text{dzRe}\left[e^{-\frac{i\omega z}{v}}E_{z}^{\text{ind}}\left(z,\omega \right)\right]$$ (7)

where *v* is the velocity of the electron, *e* is the elementary charge, ћ is the reduced Planck constant, and $E_{z}^{\text{ind}}\left(z,\omega \right)$ represents the *z*-component of the induced electric field.

In addition to electron-beam excitation, FEM simulations were also performed to compute the field enhancement and absorption cross sections of plasmonic nanostructures under optical excitation by linearly polarized light. The incident plane wave, polarized along the *x* axis, was directed normal to the sample surface. The background electric field amplitude was set to 0.5 V/nm. Scattering boundary conditions and a PML were used to absorb outgoing wave energy and prevent artificial reflections. Further details on optical simulations are provided in section S3.

### Quantum-corrected model

To capture electron tunneling across subnanometer gaps, we replaced the dielectric in the interparticle gap with an effective, frequency-dependent permittivity ε_gap_(ω,*d*) that transitions smoothly from the host medium at large separations to metallic behavior at contact (*25*, *57*–*59*). The effective permittivity ε_gap_(ω,*d*) is given by

$$\begin{array}{c} \varepsilon_{\text{gap}}\left(\omega ,d\right)=\varepsilon_{m}\left(\omega \right)+\\ \left(\varepsilon_{\text{JC}}\left(\omega \right)+\frac{{\omega_{P}}^{2}}{\omega^{2}+i\omega \gamma_{p}}-\varepsilon_{m}\left(\omega \right)\right)e^{\left(-\frac{d}{l_{d}}\right)}-\frac{{\omega_{P}}^{2}}{\omega^{2}+i\omega \gamma_{p}e^{\left(-\frac{d}{l_{c}}\right)}} \end{array}$$ (8)

where $\varepsilon_{m}\left(\omega \right)$ is the relative permittivity of the surrounding medium; $\varepsilon_{\text{JC}}\left(\omega \right)$ is the tabulated Au response; $\omega_{P}$ and $\gamma_{p}$ are the plasma and damping frequencies, respectively; *d* is the local surface-to-surface distance between metal nanoparticles; $l_{c}$ is the characteristic length at which conduction-band electron tunneling becomes notable as *d* decreases, and $l_{d}$ is the characteristic length over which the gap medium’s permittivity transitions toward the metal’s background dielectric response (*58*). We used $l_{c}$ = 0.04 nm, $l_{d}$ = 0.079 nm, $ћ\omega_{P}$ = 9.06 meV, $ћ\gamma_{p}$ = 70.76 meV, and $\varepsilon_{m}\left(\omega \right)$ = 1 (*25*, *58*). In COMSOL, $\varepsilon_{\text{gap}}\left(\omega \right)$ was implemented as a user-defined material whose real and imaginary parts are analytic functions of frequency and the local gap distance *d*(*r*), where *r* denotes the radial distance from the axis of symmetry in cylindrical coordinates. This procedure bridges classical FEM electrodynamics and quantum tunneling with minimal modification to a standard Maxwell solver while preserving physical limits at large and vanishing gaps.

### 4D-STEM

4D-STEM measurements were performed using a Merlin direct electron detector (Quantum Detectors) in 1-bit continuous reading/writing mode with a frame time of 4.8 × 10^−5^ s. A virtual annular dark-field detector was implemented using the open-source Python library py4DSTEM (*60*) to reconstruct annular dark-field images from the 4D-STEM dataset.

Electrostatic field mapping was carried out using a simplified quantum mechanical model given in Eq. 9

$$E_{⊥}=-\frac{v}{e}\frac{∆p_{⊥}}{∆z}$$ (9)

where **E**_⊥_ is the projection average of electric field along the beam direction, comprising the lateral field components *E_x_* and *E_y_* (*61*). The electric field is proportional to the change in the momentum transfer ∆**p**_⊥_, which was computed by analyzing the shift of the electron beam’s center of mass. Here, ∆*z* denotes the sample thickness, *e* is the elementary charge, and ν is the electron velocity. A homogeneous thickness of 70 nm was assumed for field reconstruction. All 4D-STEM measurements were conducted in a JEOL ARM200F FEG-STEM/TEM operating at 200 kV.

### Optical spectroscopy

Reflectance spectra of Au-T-G/Au-L/SiN*_x_* were acquired using a bright-field microspectroscopy setup. The sample was imaged using a Nikon LV100 microscope with a 20× objective lens (Nikon TU Plan ELWD 20×/0.4 numerical aperture). The field of view was restricted to the region of interest by adjusting the field stop aperture. The spectral range from 400 to 1000 nm was analyzed by coupling the microscope output to a Princeton Instruments IsoPlane 160 spectrometer. The reflectance spectra were normalized to the reflectance of an unstructured Au surface within the sample.

### SERS measurements

SERS measurements were performed with an SP-2-500i(S&I) confocal Raman spectrometer using the following parameters: laser wavelengths of 533 and 488 nm; numerical aperture of 0.5, which results in a diffraction limited lateral resolution of ~500 nm; laser power of 0.25 mW; and reflected signal from the sample fed back into the spectrometer with a 1800 g/mm grating. Raman spectra were acquired between 1000 and 3000 cm^−1^ using graphene-covered SiN*_x_*, Au-T/SiN*_x_*, Au-L/SiN*_x_*, and Au-T-G/Au-L/SiN*_x_*. Details regarding SERS enhancement factor calculations are provided in section S3.

### AFM measurements

Topographical images of the samples were obtained using a Bruker atomic force microscope (AFM) operating in tapping mode. All AFM measurements were conducted under ambient conditions with no specific control over temperature or humidity (*56*, *62*–*74*).

## Supplementary Materials

### This PDF file includes:

Sections S1 to S7 Figs. S1 to S21
