# Supplementary material for: Tunneling field emission from nano-optics under electron irradiation
Source: Sci Adv. 2026 Jan 2;12(1):eady5421. doi: 10.1126/sciadv.ady5421 (PMC12758518; doi:10.1126/sciadv.ady5421)
Supplement: Supplementary file 1 — Sections S1 to S7 Figs. S1 to S21 [file sciadv.ady5421_sm.pdf]

Science Advances
AAAS

Supplementary Materials for

Tunneling field emission from nano-optics under electron irradiation

Kenan Elibol *et al.*

Corresponding author: Kenan Elibol, k.elibol@fkf.mpg.de

Sci. Adv. **12**, eady5421 (2026)
DOI: 10.1126/sciadv.ady5421

This PDF file includes:

Sections S1 to S7
Figs. S1 to S21

Section S1. Electron-beam-induced electric field calculations

The spectrally decomposed energy loss probability $\Gamma_{EELS}(\omega)$ is obtained through full-wave electromagnetic simulations based on Maxwell's equations. These simulations are conducted using the finite-element method (FEM) in the commercial electromagnetic solver COMSOL Multiphysics. In our computational framework, we model fast electrons traveling along the $\hat{\boldsymbol{z}}$ direction with a constant kinetic energy of 200 keV. The electron beam is represented as a linear current source, which is a well-established approximation for simulating the interaction of relativistic electrons with nanostructures. Consequently, the electron beam exiting the plasmonic nanoparticles is modeled as an infinitely long, cylindrical current source.

The theoretical background of these calculations has been extensively detailed in prior studies (*62-64*). Here, we provide a brief overview of the computational approach. The spectral current density of the electron beam is defined as (1):

$$\boldsymbol{j}(z,\omega) = -e\hat{\boldsymbol{z}}\delta[\boldsymbol{R}-\boldsymbol{R}_0]e^{i\omega z/v} \tag{1}$$

where $\boldsymbol{R}_0 = (x_0, y_0)$ represents the impact parameter, and ω is the angular frequency. For an electron traversing the trajectory $\mathbf{r_e}(t)$, the energy loss is given by (2):

$$W = e\int dt\boldsymbol{v}.\boldsymbol{E}^{ind}[\boldsymbol{r}_e(t),t] \tag{2}$$

Here, v denotes the electron velocity, and $\boldsymbol{E}^{ind}(\mathbf{r},t)$ is the electric field induced by the electron beam as it interacts with a plasmonic target. In the absence of a target, a moving electron generates a background electric field, $\boldsymbol{E}_0(\mathbf{r},t)$. By spectrally decomposing equation (2), the energy loss is expressed as (3):

$$W = e\int \hbar\omega\Gamma_{EELS}(\omega)d\omega \tag{3}$$

where e is the electron charge, and $\hbar$ is the reduced Planck constant. The energy loss probability is then given by (4):

$$\Gamma_{EELS}(\omega) = \frac{ve}{2\pi\hbar\omega}\int dz Re\left[e^{-\frac{i\omega z}{v}}E_z^{ind}(z,\omega)\right] \tag{4}$$

Here, $E_z^{ind}(z,\omega)$ represents the z-component of the induced electric field. By analyzing this induced field along the source current, the energy loss probability can be extracted. The total electric field in the simulation is expressed as (5):

$$\boldsymbol{E}(\mathbf{r},\omega) = \boldsymbol{E}_0(\mathbf{r},\omega) + \boldsymbol{E}^{ind}(\mathbf{r},\omega) \tag{5}$$

In the FEM simulations, the electron beam is modeled as a cylindrical source with a diameter of 1 nm, corresponding to the approximate transverse size of the electron probe. The current density of the beam is assumed to be uniform in the transverse (*x*,*y*) plane. The entire computational domain is enclosed within a spherical perfectly matched layer (PML) of 50 nm thickness, ensuring the absorption of outgoing electromagnetic waves and preventing artificial reflections at the domain boundaries.

To compute the electron-beam-induced electric field, the background electric field $\boldsymbol{E}_0(\mathbf{r}, t)$, generated by the EELS current in vacuum, is first determined. This field is then introduced as the electromagnetic source in a simulation domain containing the plasmonic target. The induced electric field is finally obtained as (6):

$$\boldsymbol{E}^{ind}(\mathbf{r}, \omega) = \boldsymbol{E}(\mathbf{r}, \omega) - \boldsymbol{E}_0(\mathbf{r}, \omega) \tag{6}$$

This induced field is subsequently used to compute $\Gamma_{EELS}(\omega)$ as defined in equation (4).

Section S2. SERS enhancement factor

The electromagnetic enhancement factor (EF) in SERS can reach values as high as ~10^{11} within the junctions of coupled plasmonic particles. The EF is defined as equation (7):

$$EF = (I_{SERS}/I_{Raman})\times(N_{SERS}/N_{Raman}) \tag{7}$$

where I_{SERS} and I_{Raman} are the intensities of the SERS and standard Raman signals, respectively, and N_{SERS} and N_{Raman} represent the number of molecules contributing to the SERS and standard Raman signals. To determine I_{Raman}, we measure the intensity of the 2D Raman band of graphene deposited on a SiN_x substrate. Similarly, I_{SERS} is extracted from the intensity of the 2D Raman band of graphene supported on Au-T/SiN_x, Au-L/SiN_x, Au-T-G/Au-L/SiN_x structures. The estimation of N_{Raman} is based on the graphene-covered area within the laser spot size, assuming uniform molecular coverage. In contrast, the number of molecules contributing to the SERS signal N_{SERS} is structure-dependent due to the localized nature of the electromagnetic field enhancement. The N_{SERS} is determined in different systems as follows: *Au-T/SiN$_x$ system*: In this configuration, the field enhancement is primarily concentrated at the tip and edges of the Au-T emitter. The value of N_{SERS} is estimated by calculating the total surface area at these locations, assuming an Au-T tip radius of 15 nm. *Au-L/SiN$_x$ system*: Here, N_{SERS} is determined by summing the total surface area of Au grains, which exhibit an areal density of 0.5/1000 nm^{-2}. *Au-T-G/Au-L/SiN$_x$ system*: In this configuration, the dominant field enhancement occurs at the plasmonic hotspot formed between the Au-T tip and the adjacent Au-G structure. The value of N_{SERS} is estimated from the enhanced field region extracted from simulated field maps (fig. S15). The effective hotspot area is derived from the field enhancement profile. Unlike the Au-T/SiN_x system, the contribution from the Au-T edge is negligible in this structure and is therefore excluded from N_{SERS} calculations.

To ensure direct comparability of enhancement factors across different plasmonic structures, all measurements were performed using a constant laser spot size and power.

Section S3. Electromagnetic simulations with optical excitations

The FEM is employed to solve electromagnetic wave propagation in the frequency domain, enabling precise determination of the optical response of gold nanostructures across a broad spectral range. FEM simulations are conducted to analyze both the field enhancement and absorption cross-sections of plasmonic structures under varying geometric and excitation conditions.

In the simulations, the tip radius of the Au-T structure is varied from 5 to 45 nm, while the Au-G hemispheres are modeled with a fixed radius of 8.6 nm. The thicknesses of both the Au-T and Au-L structures are set at 30 nm, and the supporting SiN_x membrane has a thickness of 40 nm. The optical properties of Au are taken from the Johnson & Christy dataset, with linear interpolation applied to ensure spectral consistency (*56*). The refractive index of SiN_x is fixed at 2.4, as its dispersion is negligible within the spectral range of interest.

The excitation source is a linearly polarized plane wave propagating normal to the structures, with its electric field vector oriented along the *x*-axis. In the simulations, the amplitude of the background electric field oscillating in the *x*-direction is set to 0.5 V/nm. To ensure accurate electromagnetic wave propagation and absorption, scattering boundary conditions and a PML are implemented. The PML functions as an absorbing boundary, preventing artificial reflections of incident and scattered electromagnetic waves. To optimize wave absorption, the PML is meshed using a swept meshing approach, with five elements distributed across its thickness. The remaining simulation domain is meshed using the predefined "Finer" automatic mesh setting in COMSOL Multiphysics, balancing computational efficiency with numerical accuracy.

Beyond investigating the electric field distributions induced by optical excitation, FEM simulations are also utilized to estimate the absorption cross-sections of Au-T structures on different substrates. These simulations provide insights into substrate-dependent plasmonic interactions and their role in enhancing local electromagnetic fields.

Section S4. Absorbed beam current

The penetration depth (electron range) *(66)*, which is the average distance before an electron comes to rest, is approximated by

$$R \approx \left(\frac{0.1}{\rho}\right)(\mathrm{E_0})^{1.35} \tag{8}$$

where ρ is the specimen density ($g.cm^{-3}$) and E_0 is the primary electron energy of 200 keV. Using equation (8), the penetration depths for Au (ρ= 19.32 $g.cm^{-3}$ *(67)*) and SiN_x (ρ= 3.1-3.2 $g.cm^{-3}$ *(68)*) are calculated to be 6.62 µm and 39.92-41.21 µm.

In a thin sample, the absorbed beam current (I_A) is approximated by $I_A/I_B = t/R$, where *t* is the thickness of the sample and I_B is the incident electron beam current *(69)*. For our sample, comprising 60 nm Au (30 nm triangles on a 30 nm layer) on top of a 40 nm SiN_x, the fraction of total absorbed current is ~1.00–1.01%. In other words, ~1% of the incident electrons are stopped while ~99% are transmitted (elastically or inelastically). Thus, the absorbed beam current is negligible in our samples.

Section S5. Plasmon energy transfer to secondary electrons

Plasmon-mediated energy transfer plays a pivotal role in enhancing SE emission from gold, despite its relatively high work function (W_F = 4.9 eV) exceeding its characteristic plasmon energy (~2.3 eV) (*65*). Upon decay, surface plasmons can transfer energy to conduction electrons, generating hot carriers that contribute to SE emission. However, since the plasmon energy of gold is insufficient to directly overcome the work function, alternative energy gain mechanisms such as plasmon-assisted electron-electron scattering and sequential plasmon absorption become essential.

Recent studies have demonstrated that SEs emitted near the vacuum level can interact with surface plasmons in close proximity to the gold surface, gaining an energy of ~2.3 eV before emission (*65*). This plasmon-induced energy gain manifests as distinct peaks in SE emission spectra, establishing a direct correlation between plasmon dynamics and SE generation. The efficiency of this process is governed by factors such as plasmon lifetime, mode confinement and surface properties. Notably, polycrystalline gold surfaces exhibit stronger SE emission enhancement compared to smooth single-crystal surfaces, owing to increased plasmonic interactions. Additionally, the interaction time between SEs and LSPRs is extended due to the relatively low velocity of SEs, thereby increasing the probability of energy transfer from plasmons to SEs.

Section S6. Secondary electron generation by plasmon decay

Plasmon decay occurs through two primary mechanisms: radiative and non-radiative pathways. In the radiative decay process, plasmons couple to free-space photons, leading to scattering and far-field emissions (*70*). In contrast, non-radiative decay channels – such as Landau damping and interband transitions – facilitate energy transfer from plasmons to individual electrons within the metal, exciting them into higher-energy states (*3*). If these hot electrons acquire sufficient energy, they can overcome the material's work function and escape into free space as SEs (*71*). Understanding this process is critical for optimizing SE yield in plasmon-enhanced electron emission and for refining energy dissipation pathways in plasmonic nanostructures. Plasmon decay can directly excite SEs if the plasmon energy exceeds the work function of the target material, following the relation ($E_{SE} = \hbar\omega - W_F$), where E_{SE} is the kinetic energy of the emitted SE, $\hbar\omega$ is the plasmon energy, and W_F is the work function (*51*, *72*, *73*). For efficient SE generation, hot electrons produced by non-radiative plasmon decay must attain sufficient energy to surpass the work function of the emitting material (*51*, *72*, *73*). However, in the case of gold, individual plasmon resonances lie below 2.3 eV, which is significantly lower than its work function (W_F = 4.9 eV). Consequently, a single plasmon decay event is insufficient to directly induce SE emission in Au. Werner *et al.* proposed that SE emission in this context follows a mechanism analogous to photoelectron emission, where plasmon decay effectively replaces photon absorption as the energy source (*51*). This process is particularly relevant for materials whose LSPR energies exceed their work functions, such as aluminum, where efficient SE emission via plasmon decay can be realized.

Section S7. Hot-electron limitations

Hot-electron emission into the vacuum via plasmon decay occurs when surface plasmons transfer their energy to conduction electrons, enabling them to overcome the material's work function and escape into free space (*52*). As discussed previously, this process is driven by non-radiative plasmon decay mechanisms, where plasmon energy is absorbed by individual electrons, promoting them to higher-energy states. If these hot electrons are generated within their mean free path (typically < 10 nm in metals), they can reach the surface with minimal energy loss and be emitted into the vacuum. However, since the plasmon energy of gold (~2.3 eV) is lower than its work function (W_F = 4.9 eV), direct hot-electron emission is energetically unfavorable.

In contrast, SEs are significantly more abundant because they originate from direct inelastic scattering of the 200 kV electron beam with a large number of conduction and core electrons. Unlike hot-electron emission, SE generation is less dependent on the material's plasmonic properties and occurs more uniformly across different surfaces. Hot-electron emission, however,

is strongly influenced by the excitation and efficient decay of LSPRs and SPPs, which require specific geometric and material conditions (*74*). The efficiencies of LSPR and SPP excitations as well as their subsequent decay into hot electrons are affected by several factors, including surface roughness, film thickness and the presence of nanostructures (*74*). Consequently, the yield of hot-electron emission is typically lower than that of SEs, as not all plasmonic decays result in hot-electron generation, and the process is inherently constrained by the plasmonic efficiency of the material.

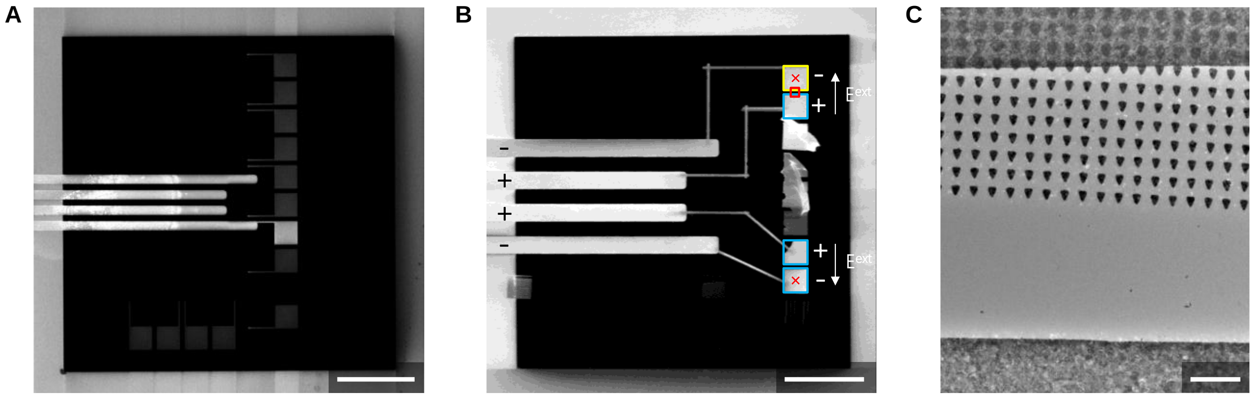

Fig. S1. Samples used for in-situ biasing experiments in TEM. (**A** and **B**) HAADF-STEM images of the sample chips including large electrodes patterned by electron beam lithography. In (**B**), the area marked in the yellow frame contains Au-T-G/Au-L/SiN_x while the areas within the blue frames show Au-L/SiNx. The areas exposed to the electron beam are marked by a red ×. (**C**) Close-up TEM image of the area marked by the red frame in (**B**). Scale bars: 50 μm (**A**,**B**) and 0.5 μm (**C**).

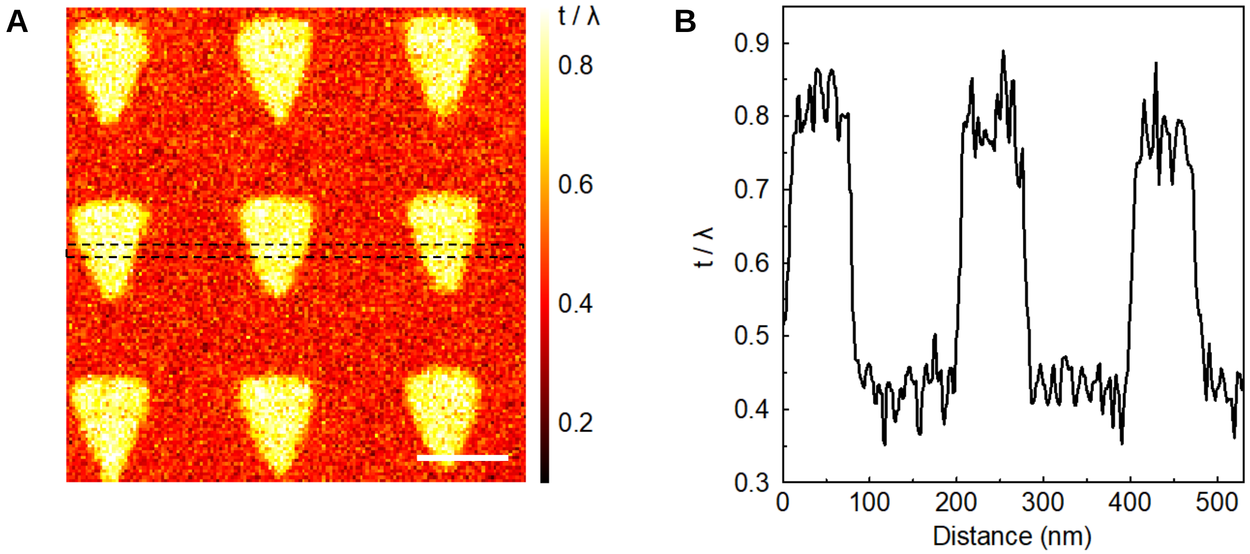

Fig. S2. Au-T thickness. (**A**) The t/λ map for Au-T/SiN_x. (**B**) The line profile recorded along the black dashed frame in (**A**). Scale bar: 100 nm (**A**).

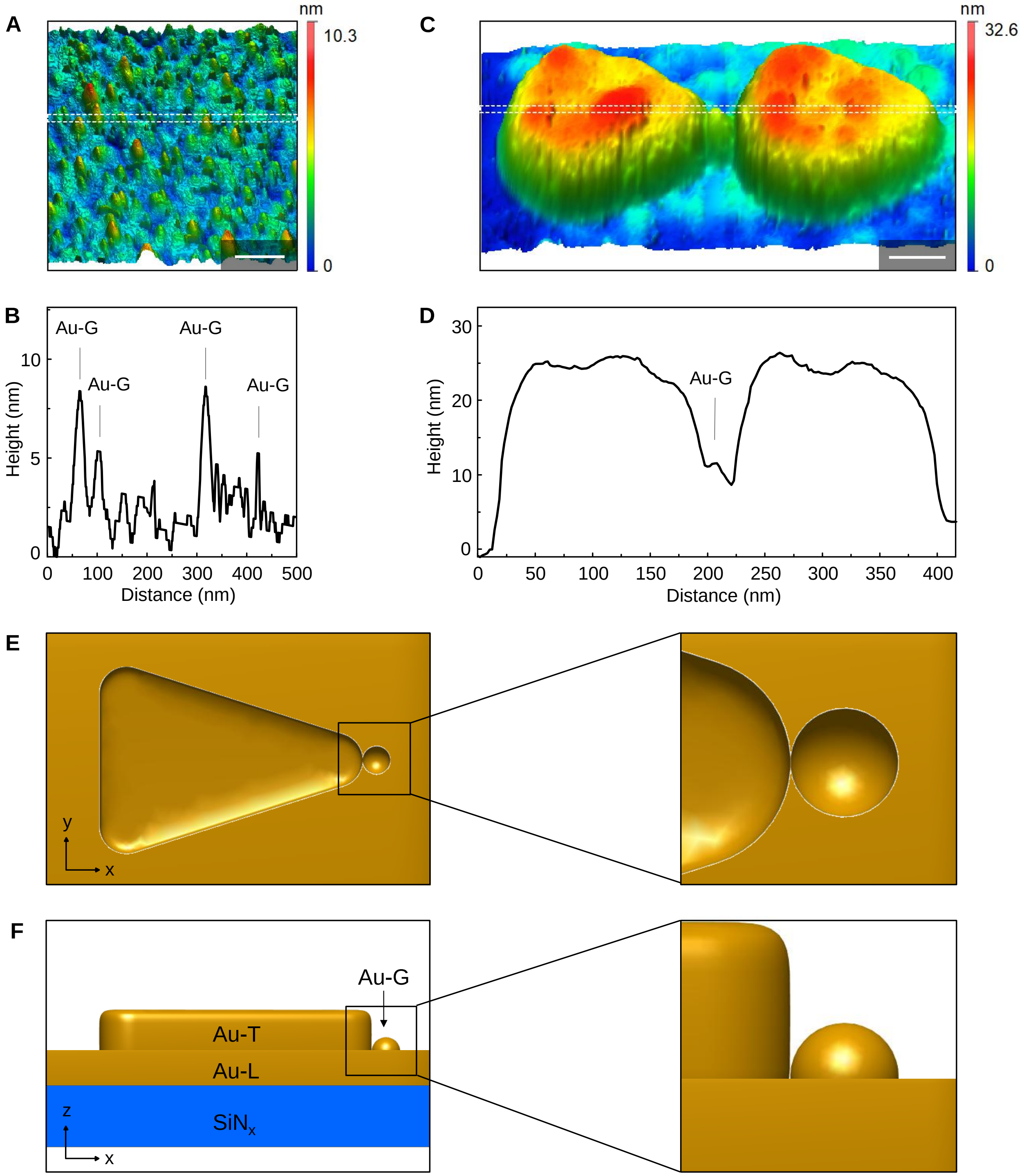

Fig. S3. Gold grains coupled to Au-T. (**A** and **B**) 3D AFM topography image for Au-L/SiN_x and the height profile recorded along the dashed white box on (**A**). (**C** and **D**) 3D AFM topography image for Au-T-G/Au-L/SiN_x and the height profile recorded along the dashed white box on (**C**). (**E**, **F**) Plane and side views of a model with an Au-T-G/Au-L/SiN_x. Scale bars: 100 nm (**A**) and 50 nm (**C**).

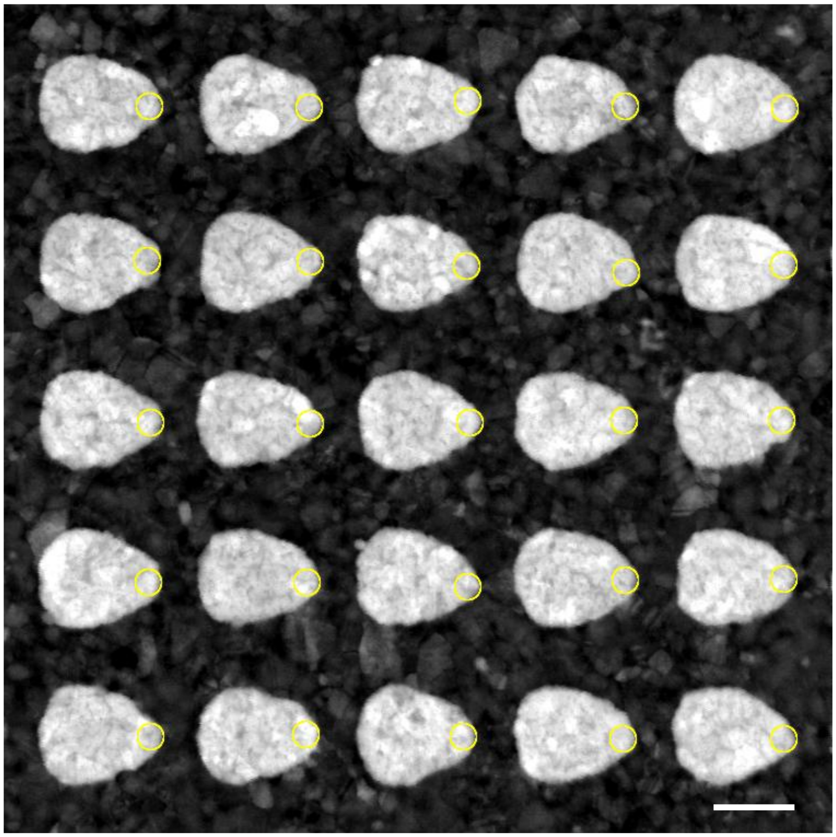

Fig. S4. Tip radii of the Au triangles. Tip radii of the Au triangles were obtained by fitting a circle to the apex boundary. Before fitting, the HAADF–STEM images were denoised with an edge-preserving filter to retain sharp edges. The yellow circles are fits to the tips of Au-Ts. Scale bar: 100 nm.

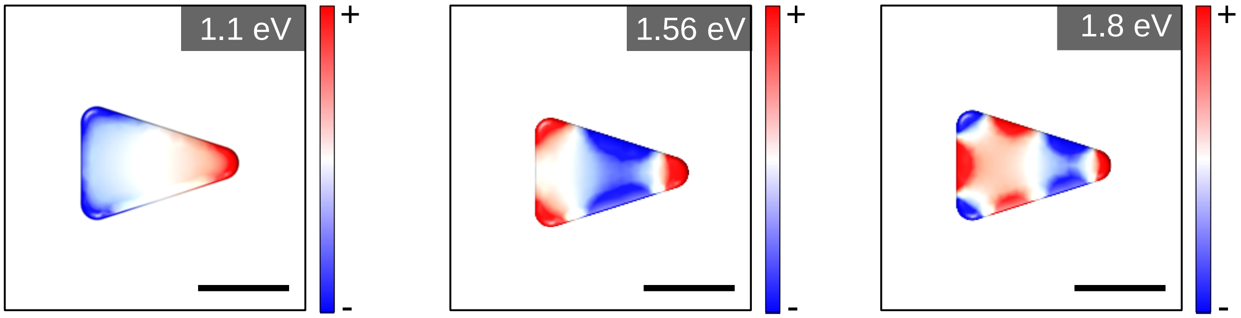

Fig. S5. LSPRs excited in Au-T/SiN$_x$. Surface eigencharge distributions showing dipole (at 1.1 eV) and edge (at 1.56 and 1.8 eV) LSPRs excited in Au-T/SiN$_x$. The surface eigencharge was obtained at the energies identified from the EEL spectra in Fig. 1F. Scale bars: 100 nm.

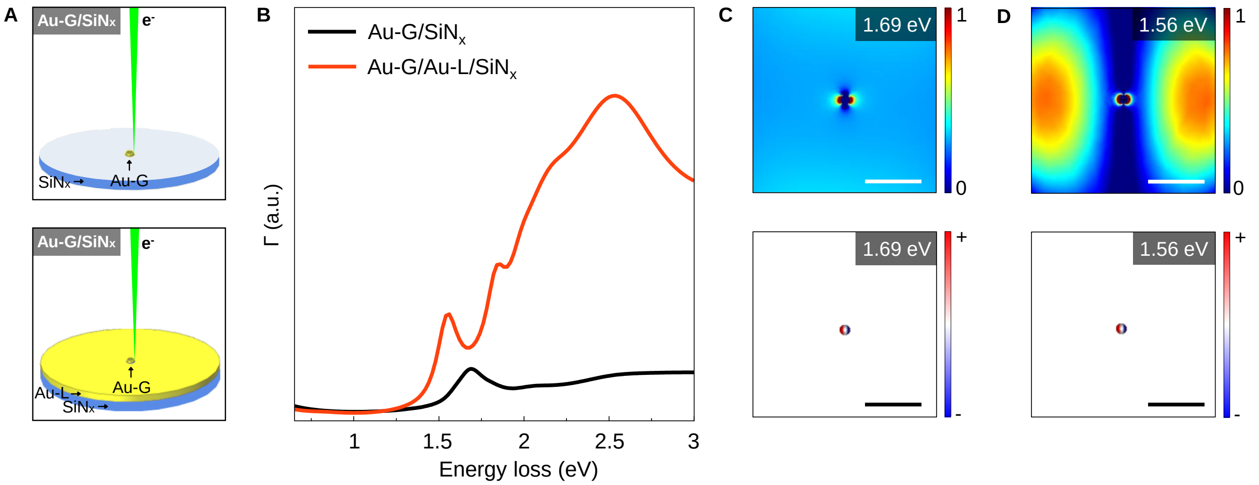

Fig. S6. Substrate effect on Au-G. (**A**) Schematics of Au-G/SiN_x and Au-G/Au-L/SiN_x. (**B**) Simulated EEL spectra for Au-G/SiN_x and Au-G/Au-L/SiN_x. The EEL spectra are derived from the edge of the Au hemisphere. (**C** and **D**) Simulated normalized electric field maps and surface eigencharge distributions obtained at the energies identified from the simulated EEL spectra in (**B**). Scale bars: 100 nm (**C**, **D**).

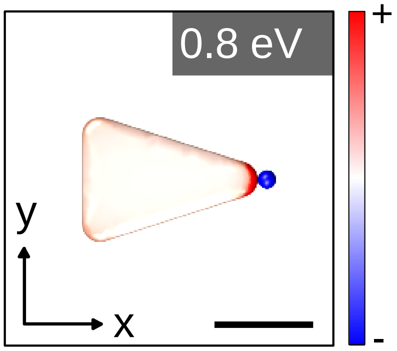

Fig. S7. Charge transfer plasmon. The surface eigencharge distribution obtained at 0.8 eV for Au-T-G/Au-L/SiN_x structure.

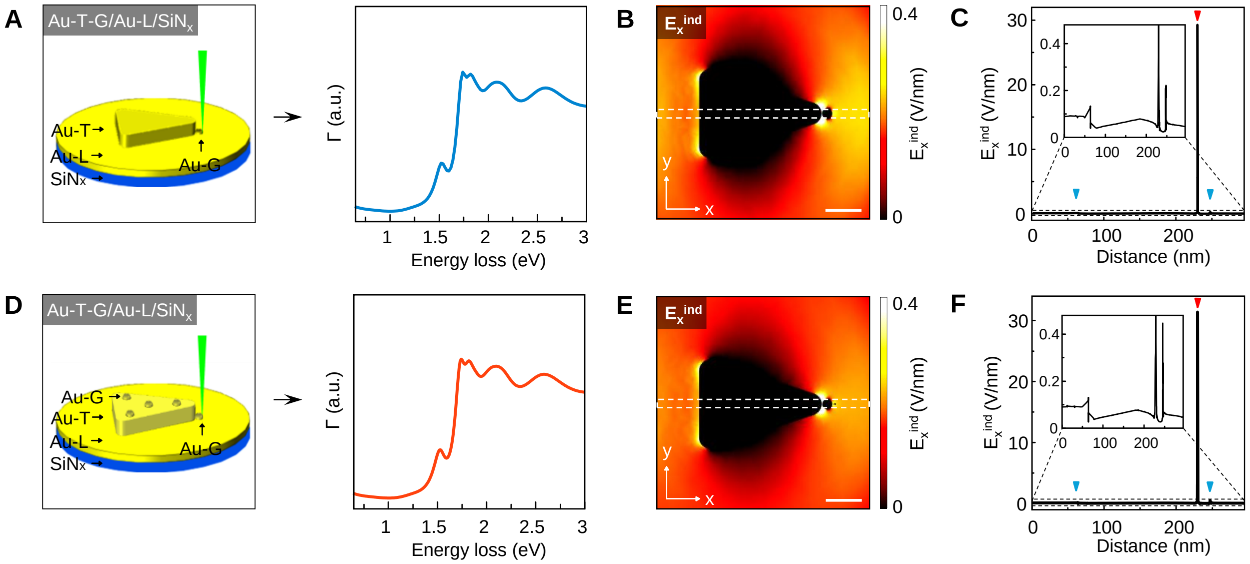

Fig. S8. Comparison between the ideal and non-ideal structures. (**A**) Schematic of the Au-T/SiN_x and its corresponding EEL spectra. (**B** and **C**) Electron-beam-induced electric field (E^{ind}) map computed at the dipole energy and the corresponding electric field profile recorded along the white frame in (**B**). (**D**) Schematic of the Au-T/SiNx with Au grains on Au-T and its corresponding EEL spectra. (**E** and **F**) Electron-beam-induced electric field (E^{ind}) map computed at the dipole energy and the corresponding electric field profile recorded along the white frame in (**E**). Scale bars: 50 nm (**B**, **E**).

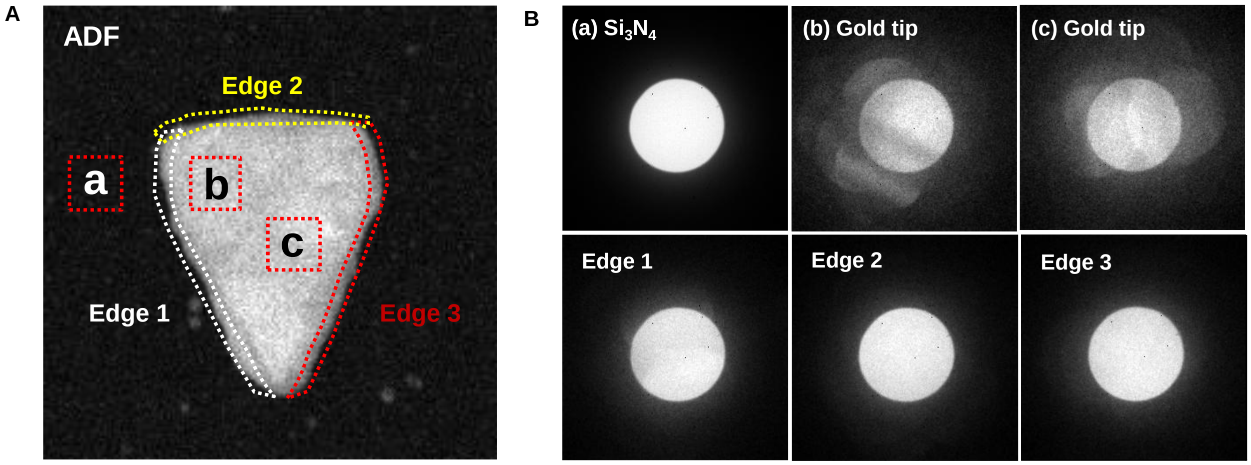

Fig. S9. Position-averaged convergent-beam electron diffraction. (**A**) ADF image of a Au triangle (Au-T). (**B**) PACBED patterns extracted from the interior and the edge regions of the Au-T. PACBED images are obtained from the areas marked on (**A**).

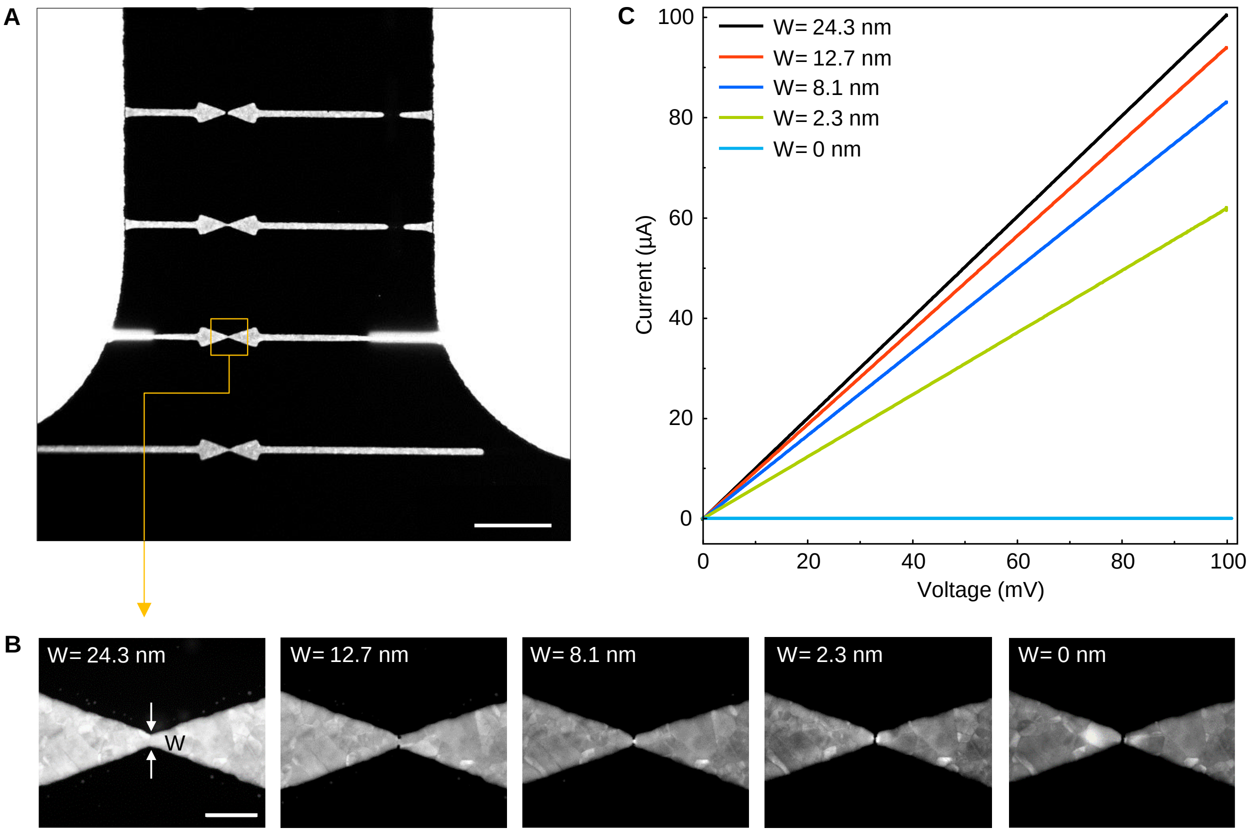

Fig. S10. Nanogap formation within the nanostructures. (**A**) HAADF-STEM image of bowties on SiN_x membrane. (**B**) Close-up HAADF-STEM image of a gold nanostructure with different junction widths modified by a focused electron beam. (**C**) The current-voltage measurements for the coupled gold triangles with different junction widths. Scale bars: 1 μm (**A**) and 100 nm (**B**).

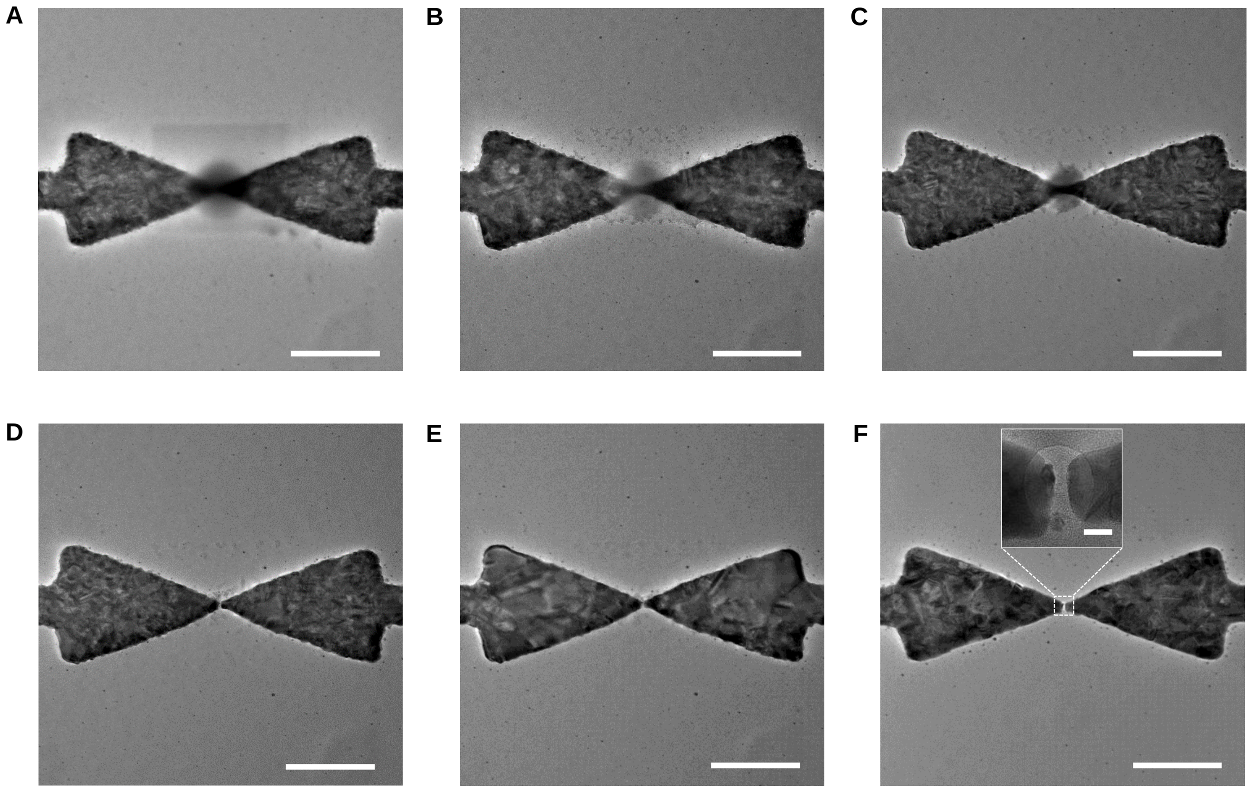

Fig. S11. Cleaning hotspots. TEM images of coupled triangles with an electron-beam–induced gap. The images were taken after each O_2 plasma treatment with different recipes. The recipes used for O_2 plasma treatment are given as follows: (**A**) Mixed gas rate: O_2/Ar (5:95), power: 80%, gas flow: 30 sccm, time: 2 min. (**B**) Mixed gas rate: O_2/Ar (10:90), power: 80%, gas flow: 30 sccm, time: 3 min. (**C**) Mixed gas rate: O_2/Ar (10:90), power: 90%, gas flow: 30 sccm, time: 3 min. (**D**) Mixed gas rate: O_2/Ar (15:85), power: 95%, gas flow: 30 sccm, time: 4 min. (**E**) Mixed gas rate: O_2/Ar (30:70), power: 95%, gas flow: 30 sccm, time: 4 min. (**F**) Mixed gas rate: O_2/Ar (40:60), power: 95%, gas flow: 30 sccm, time: 15 min. Scale bars: 200 nm (**A**-**F**), 10 nm (inset of **F**).

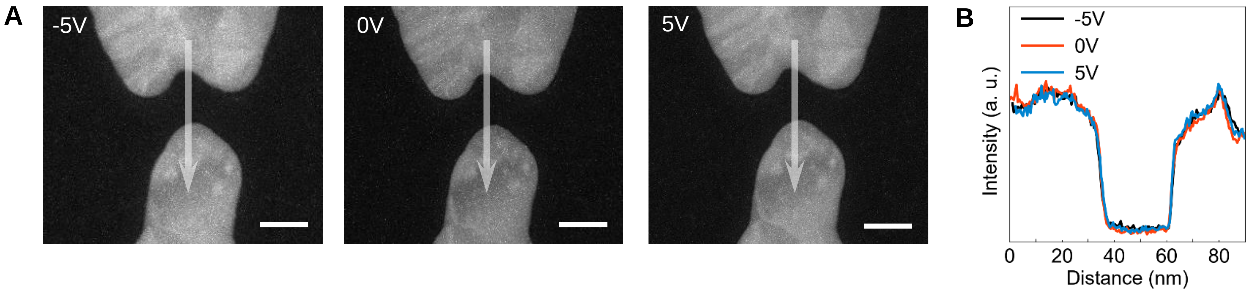

Fig. S12. Sample morphology at different biases. (**A**) HAADF-STEM images of a hotspot recorded when the applied bias is -5, 0 and 5 V. (**B**) Line profile recorded along the semitransparent white line in (**A**). Scale bar: 50 nm (**A**).

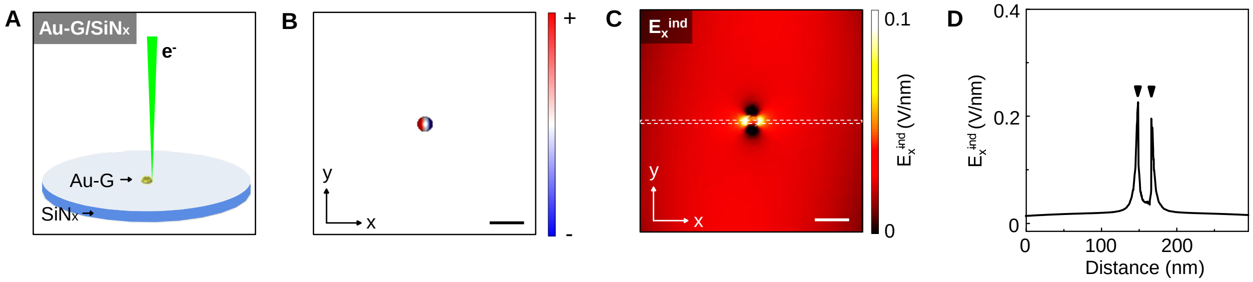

Fig. S13. Electron-beam-induced electric field on Au-G. (**A**) Schematic of the Au-G/SiN_x model used in FEM simulations. (**B**) Surface eigencharge distribution calculated at the dipole energy of the Au grain with a radius of 8.6 nm. (**C**) Computed electron-beam-induced electric field map derived at the dipole energy. (**D**) The electric field strength recorded along the white frame in (**C**). Scale bars: 50 nm (**B** and **C**).

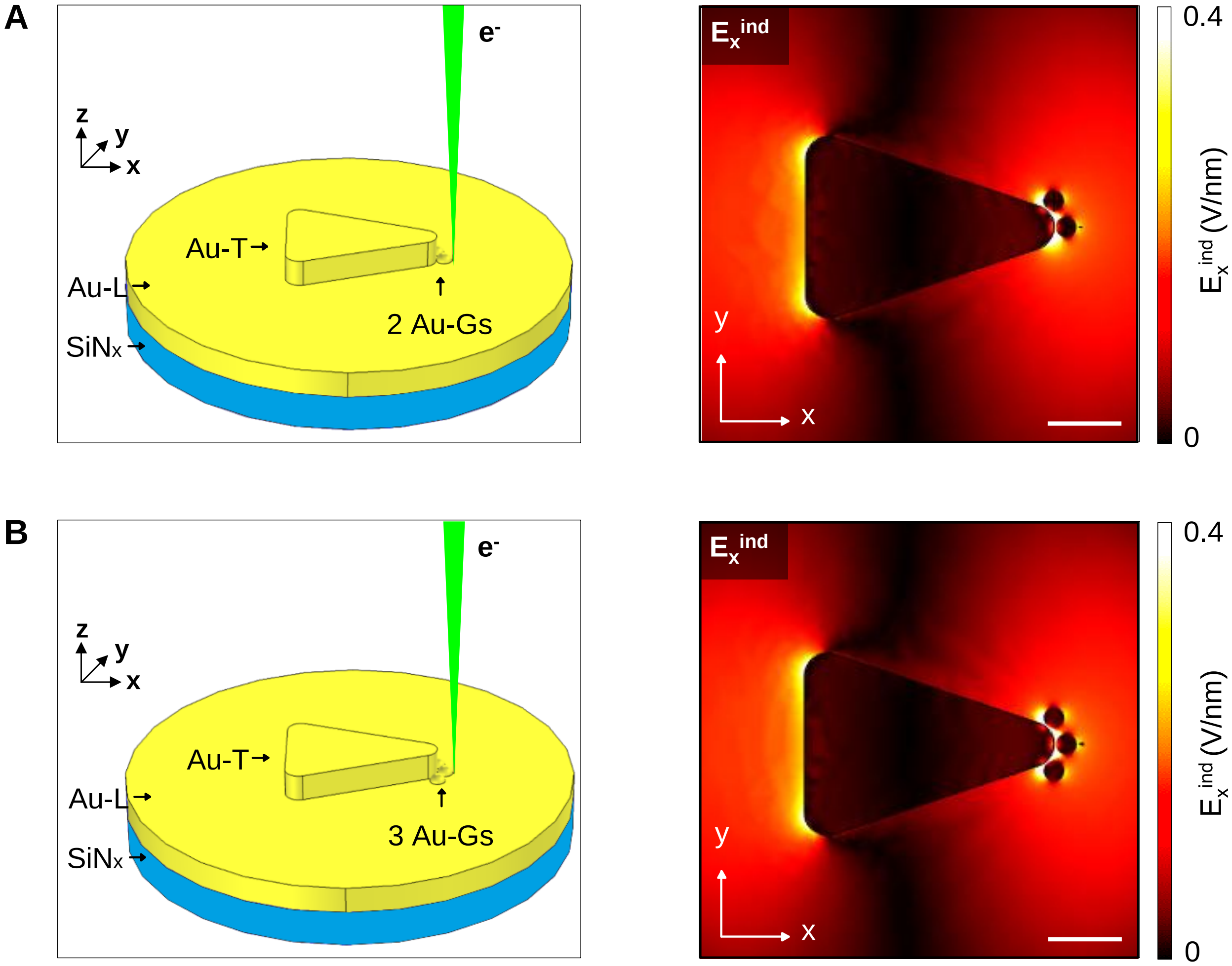

Fig. S14. Au-T coupled to several Au-Gs. (**A** and **B**) Schematic of Au-T-G/Au-L/SiN_x with two and three Au-Gs and corresponding computed electron-beam-induced electric field (E_x^{ind}) maps derived at the dipole energy. Scale bars: 50 nm (**A** and **B**).

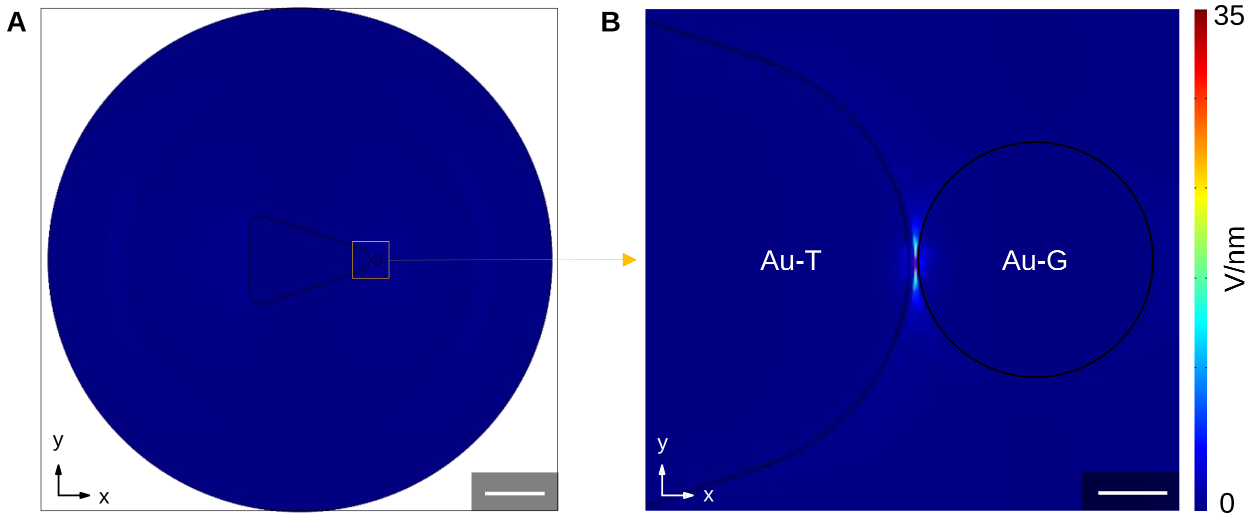

Fig. S15. Estimation of the gap area. Electric field distributions along the *x*-axis of Au-T-G/Au-L/SiN_x, which is excited optically. Scale bars: 100 nm (**A**) and 5 nm (**B**).

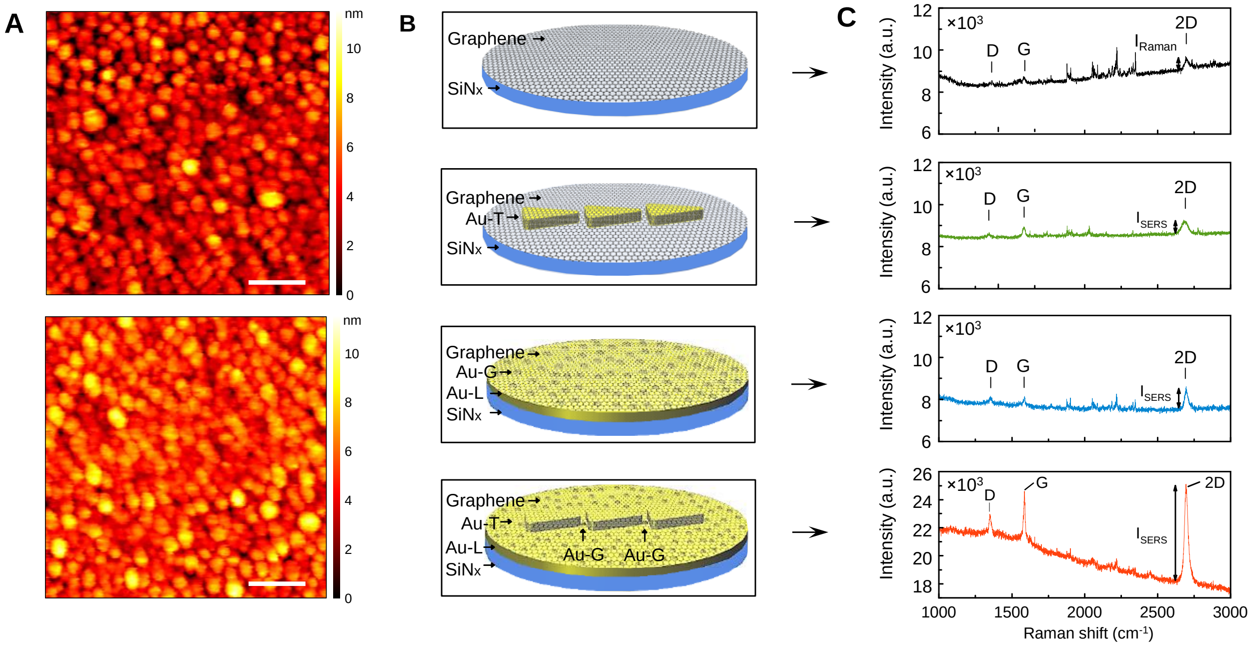

Fig. S16. SERS measurements with an additional control experiment. (**A**) AFM topography image of Au grains formed on a SiN_x membrane. The grains were produced by depositing a 2 nm thick Au layer on SiN_x followed by annealing at 160 °C. (**B**) Schematic representation of the monolayer graphene-coated structures used in SERS measurements, including SiN_x, Au-T/SiN_x, Au-L/SiN_x, and Au-T-G/Au-L/SiN_x. (**C**) Raman spectra of monolayer graphene deposited on the substrates illustrated in (**B**). Scale bars: 100 nm (**A**).

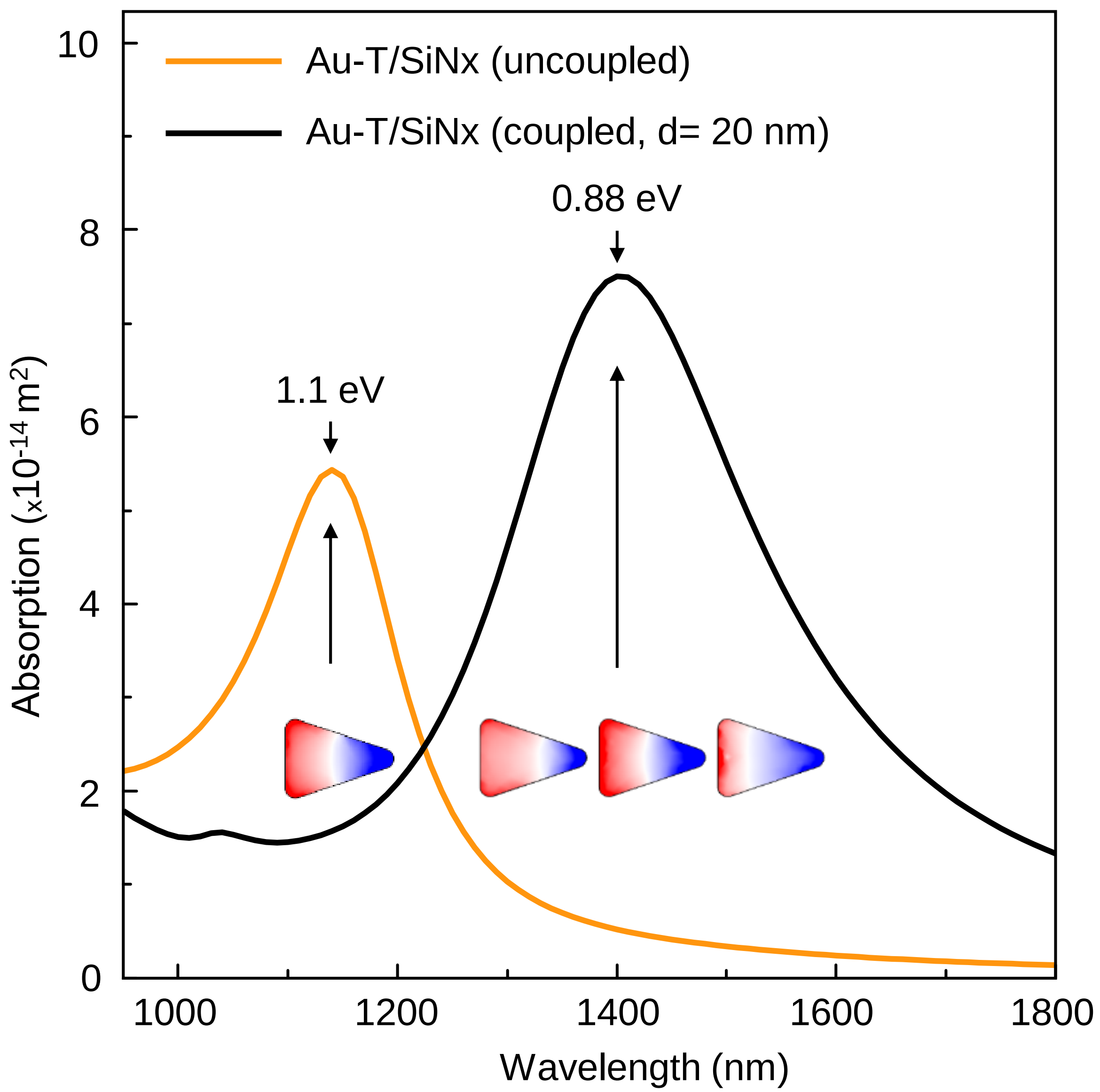

Fig. S17. Absorption spectra for coupled and uncoupled Au-Ts. (**A**) Calculated absorption spectrum for uncoupled and coupled Au-Ts on SiN_x.

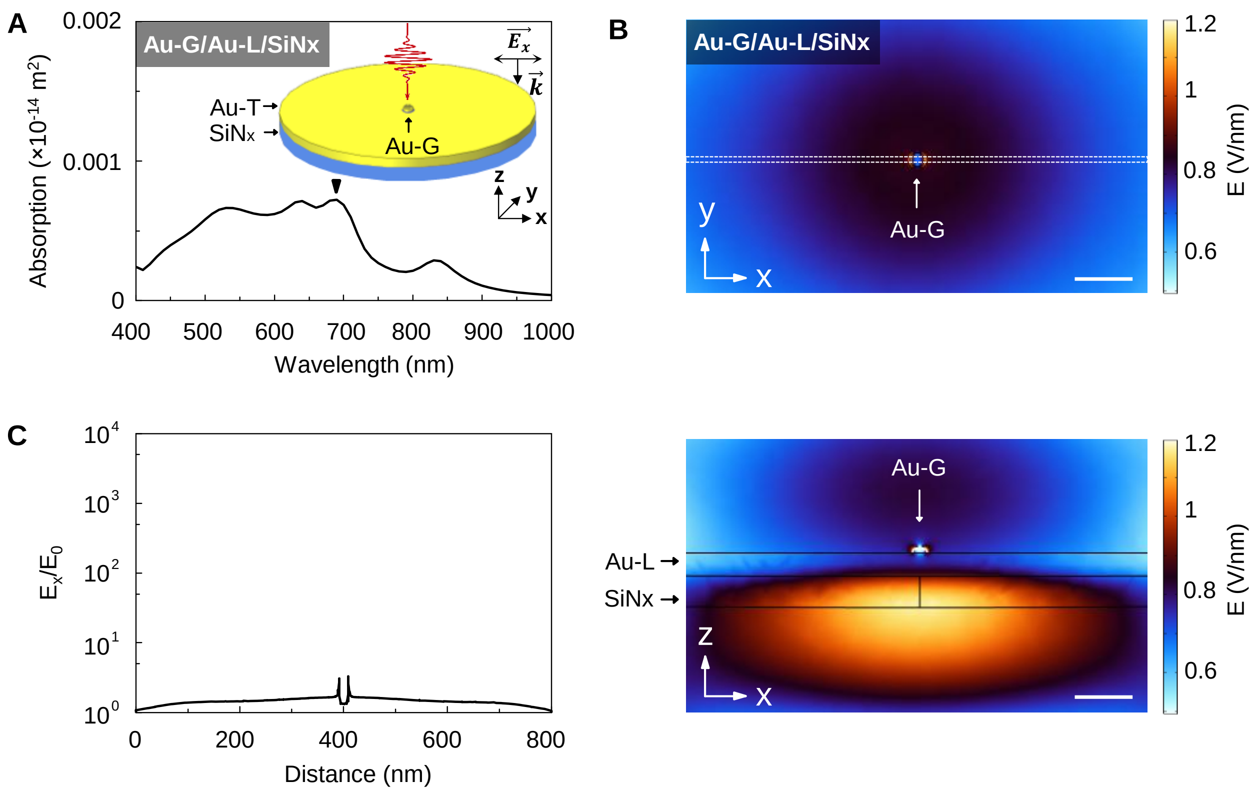

Fig. S18. Field enhancement on Au-G. (**A**) Calculated absorption spectra for Au-G/Au-L/SiN_x. The dipole peak is marked with a black arrow. The inset shows the schematic of the model used in the FEM simulations. (**B**) Simulated electric field distributions along the *x*-axis. The electric field map is generated at the dipole energy of the Au-G. (**C**) Field enhancement profile recorded along the white dashed frame in (**B**). Scale bars: 100 nm (**B**).

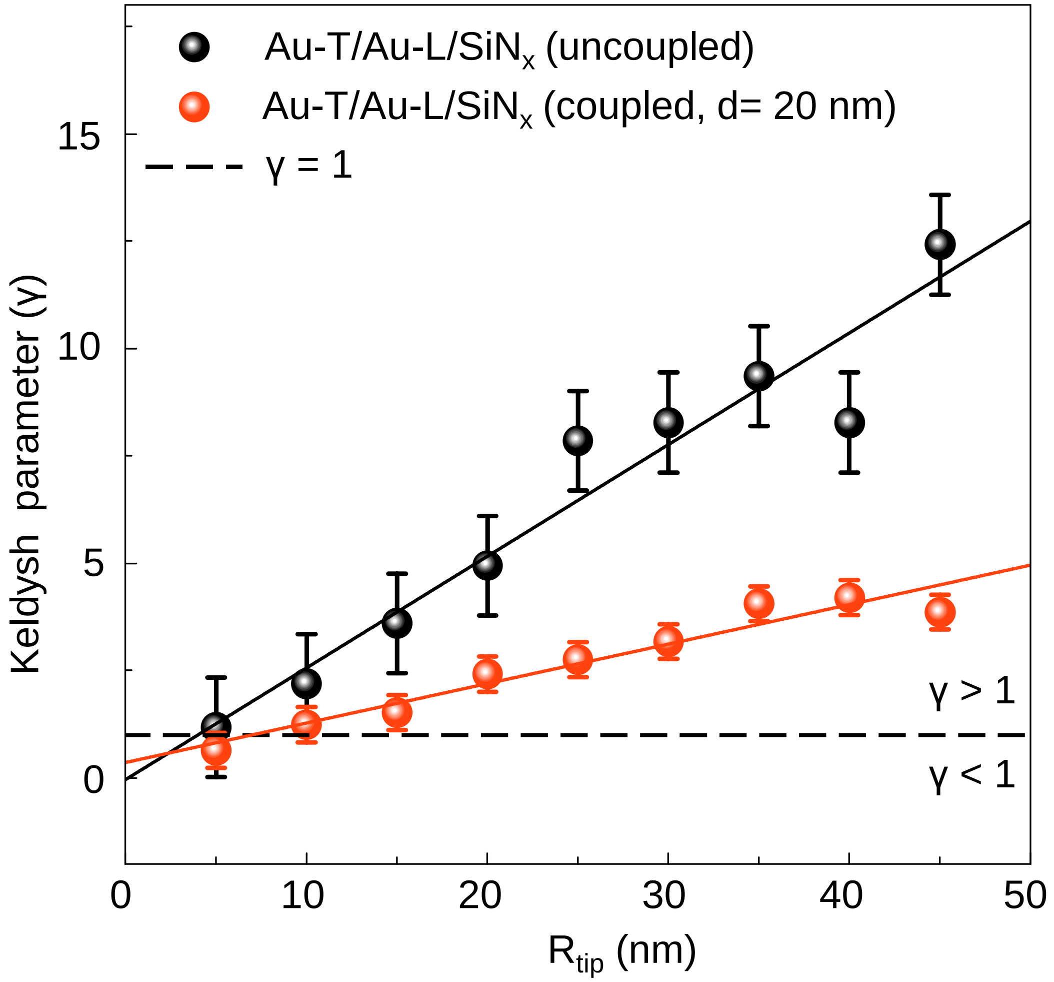

Fig. S19 Keldysh parameters. Keldysh parameters calculated for the Au-T/Au-L/SiN_x (no coupling between the Au triangles) and Au-T/Au-L/SiN_x (Au triangles are coupled because the distance (d) between the Au triangles is 20 nm). The tip radius is varied from 5 to 45 nm. Error bars indicate the standard error of the mean.

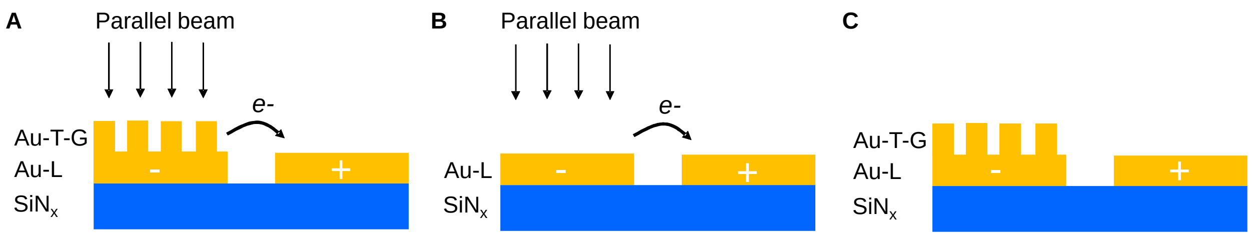

Fig. S20. Schematic of beam positions and sample configurations. (**A-C**) Configurations used to measure $I_{total} = I_{FE} + I_{SE} + I_{BC}$, I_{SE}, and I_{BC}, respectively. Samples in (**A**) and (**B**) are illuminated with a parallel electron beam while the sample in (**C**) is measured without electron-beam illumination.

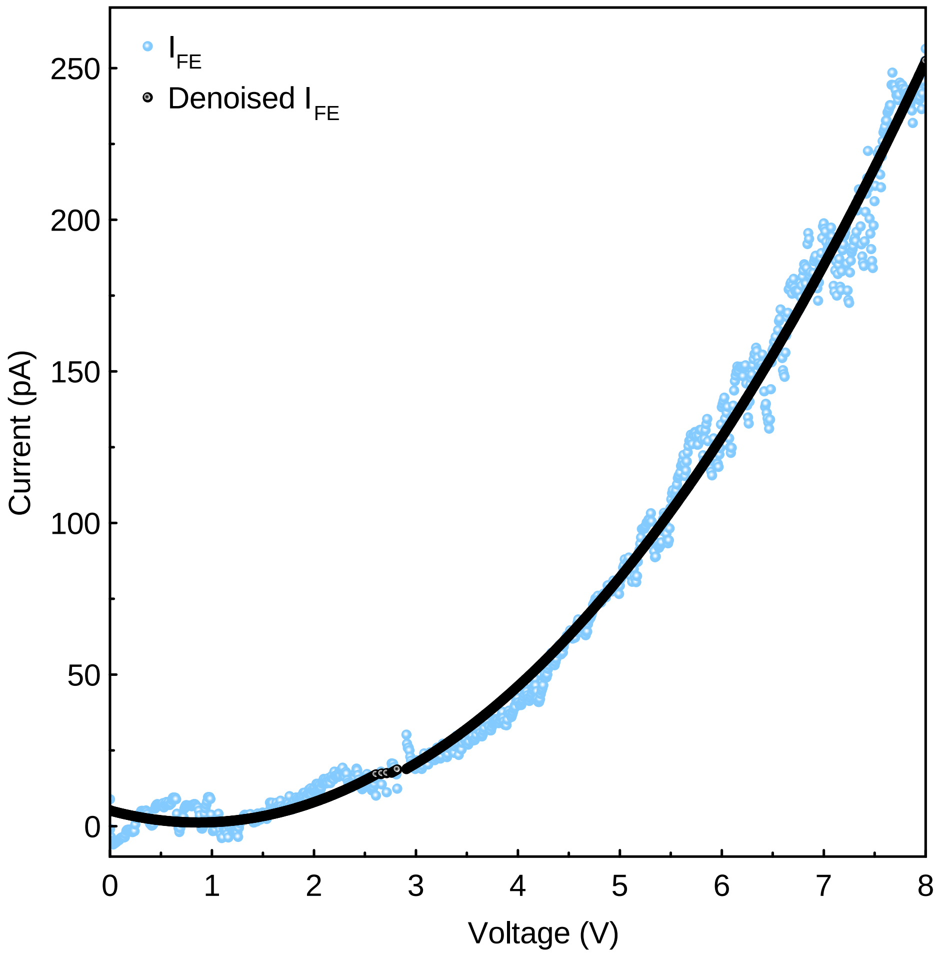

Fig. S21. Denoising of experimental current-voltage data. Raw experimental data and data denoised by Savitzky-Golay filter smoothing based on a quadratic polynomial.
